# Biodegradable Polymeric Core/Shell Nanoformulations Encapsulating Essential Oils: Physicochemical Design, Controlled Release, and Targeted Acne and Sebum Management

**DOI:** 10.3390/polym18050621

**Published:** 2026-03-01

**Authors:** Weronika Syryczyk, Kamila Bedkowska, Maria Pastrafidou, Antonis Avranas, Ioannis A. Kartsonakis

**Affiliations:** 1 Institute of Chemical Sciences, Faculty of Chemistry, Maria Curie-Sklodowska University, Maria Curie-Sklodowska Sq. 3, 20031 Lublin, Poland; weronika.syryczyk@o2.pl (W.S.); kamilabedkowska25@gmail.com (K.B.); 2 Laboratory of Physical Chemistry, School of Chemistry, Aristotle University of Thessaloniki, 54124 Thessaloniki, Greece; mpastraa@chem.auth.gr (M.P.); avranas@chem.auth.gr (A.A.)

**Keywords:** biodegradable polymers, core/shell polymers, eco-friendly polymers, nanoformulations, essential oils, acne, sebum, physical chemistry

## Abstract

This review examines biodegradable polymer-based core–shell nanoformulations encapsulating essential oils for acne treatment through the lens of physicochemical design and controlled delivery mechanisms. Acne is a common inflammatory skin disorder closely associated with sebum overproduction and microbial imbalance, while conventional therapies, although effective, may present long-term side effects. Increasing attention has therefore turned to sustainable dermatological materials derived from eco-friendly polymers combined with naturally active compounds. Recent advances show that core–shell nanostructures fabricated from biodegradable polymers function as physicochemically engineered carriers for volatile essential oils. They enhance their stability and protect them from premature degradation. They also enable controlled release governed by diffusion, polymer relaxation, interfacial interactions, and degradation kinetics. This review highlights how polymer chemistry, interfacial properties, particle morphology, and processing routes determine encapsulation efficiency, release profiles, and skin permeation behaviour. Particular emphasis is placed on structure–property–function relationships, including mass transport phenomena, thermodynamic compatibility between polymers and essential oils, surface charge, wettability, and nanostructure architecture, which collectively influence bioavailability and therapeutic performance. By integrating concepts from polymer physical chemistry, colloid and interface science, and drug delivery kinetics, these sustainable nanoformulations emerge as promising platforms for acne and sebum control. Overall, essential oil-loaded biodegradable polymeric core–shell systems represent a sustainable and scientifically grounded approach to acne management, although further physicochemical characterization, in vivo validation, and consideration of cost, technical challenges, and current limitations are required to support clinical translation.

## 1. Introduction

Acne is an inflammatory skin disorder involving follicular hyper-keratinization, microbial colonization, inflammation and excessive sebum production. Sebum is a key factor, promoting *Cutibacterium acnes* proliferation and contributing to oxidative stress and inflammatory cascades within pilosebaceous units.

The study by Apostolos Pappas et al. (2009) [1] intentioned to detect which lipid components of sebum could be related to the tendency to develop acne. Sebum produced by sebaceous glands contains a variety of lipids. Those lipids are mainly triglycerides, wax esters, squalene, and free fatty acids. In addition, there are also minor amounts of cholesterol-based compounds and diglycerides. Since acne usually appears in body areas rich in these glands, excessive sebum secretion is considered an important factor in the disorder’s development. In the study, sebum lipids from acne patients were compared with those from unaffected individuals. Those with acne produced more sebum overall. Free fatty acids were the only group present at lower levels, while triglycerides, wax esters and cholesterol esters were increased. The largest difference was observed for squalene, which was about 2.2 times higher in the acne group.

Moreover, research conducted by C. C. Zouboulis et al. (2005) [2] focused on important aspects in acne pathophysiology. Androgens are one of the key factors causing the development of acne. They act on the sebaceous glands, stimulating their growth, while promoting proliferation of keratinocytes. Elevated androgen levels led to higher sebum production and the appearance of potent acne lesions. Along with that, imbalances in sebaceous follicle function can be provoked by various factors. Those are, for example, peroxisome proliferator-activated receptor (PPAR) ligands, regulatory neuropeptides, and environmental influences. These factors activate inflammation and promote the evolution of pre-comedones into mature microcomedones. Another reason for acne exacerbation is Propionibacterium acnes. The species does not clearly affect the early stages of acne but may participate in a later response, which will cause progression of acne.

Typical acne treatments like retinoids, antibiotics, and synthetic antimicrobials can often cause adverse skin reactions like antimicrobial resistance and systemic toxicity. A study conducted by Weaam Said Awadh Al Yaqoubi et al. (2024) [3] explored the effects of isotretinoin on acne patients. This antibiotic is generally considered as one of the most successful medicaments for severe acne. This pharmacology option reduces sebaceous gland function, which are responsible for sebum production. It also has anti-inflammatory and immune-regulating effects, which improve its effectiveness. Researchers detected changes in liver function tests in isotretinoin patients. The results showed raises in bilirubin and alanine aminotransferase levels, which suggests hepatic stress. Analysis also included lipid profiles assay. The findings revealed rises in values of cholesterol, low-density lipoprotein, high-density lipoprotein, and triglycerides, indicating the importance of liver function and lipid metabolism monitoring during isotretinoin therapy.

In the work of Amr Abdelhamed et al. (2021) [4], isotretinoin has been shown to affect pituitary–ovarian axis function. The researchers suggest that the examined medicament may activate the forkhead box O transcription factor, p53/FOXO1, pathway. Upregulation of p53 and FOXO1 promotes apoptosis and regulates gonadotropin expression and granulosa cell homeostasis. Consequently, gonadotropin release and granulosa cell apoptosis are modified, leading to a smaller follicular reserve. This clinical study mainly focuses on menstrual changes. One study with 40 women who used isotretinoin for four months showed that menstrual irregularities appeared in 20% of participants. Similar tendencies were observed in another group of 30 patients treated for three months, where cycle disruptions appeared in one-third of the women. Researchers also examined whether isotretinoin affects broader endocrine functions or not. Three months of treatment minimized the luteinizing hormone, prolactin, and total testosterone, which are linked to some signs of thyroid and adrenal imbalance. Another clinical and experimental study also examined the drug’s impact on ovarian function. The results suggest that isotretinoin may influence not only p53/FOXO1 pathway but also other hormonal pathways, which are important for female reproductive health.

Special attention has been given to natural bioactive compounds, especially essential oils, for their antimicrobial, anti-inflammatory, and sebum-regulating properties. However, their clinical application is limited due to high-volatility, chemical instability, or potential skin irritation when used in their free form. According to Naga Parameswari Mangalagiri et al. (2021)’s research [5], one of the reasons why essential oils gain more attention is their strong antimicrobial properties. These secondary metabolites derived from plants and are known for their ability to limit the growth of different microorganisms. This feature is mainly linked to their monoterpene components. In plants, such compounds may also contribute to natural protection against diseases. In this experiment, seven essential oils from plants were tested for their antibacterial properties with the paper disc method. All of them showed activity against at least some of the bacterial strains. The strongest antibacterial effects were found in lemongrass, palmarosa, eucalyptus, and geranium essential oils. Another study (Piotr Szweda et al. (2017) [6] reported that essential oils from *Artemisia afra*, *Pteronia incana* and *Rosmarinus officinalis* show antimicrobial activity toward 41 microbial strains. These strains include bacteria and yeasts responsible for food spoilage, as well as common pathogens affecting humans and plants.

Also, a study by Eugenia Ganosi et al. (2023) [7] analyzed the selected essential oils, particularly their volatile components. Essential oils can degrade easily through chemical or enzymatic reactions. Their short- and long-term stability depends on several factors, which affect their quality. Those are, for example, their physicochemical characteristics and the environmental conditions. During this study, the essential oil samples were stored in different types of containers for up to six months and temperatures ranging from −20 °C to 45 °C. Samples were periodically analyzed using Gas Chromatography-Mass Spectrometry (GC-MS). The principal components of the essential oils changed only slightly during the first three months. The smell of the oils stayed mostly the same throughout the storage period, no matter the conditions. This happens because the main compounds are the ones giving the oils most of their scent, while the others have only a very small effect. The oils had the best quality when stored at 4 °C away from sunlight. Exposure to high temperatures or direct heat led to a clear decline in their quality [7].

Nanotechnology-based delivery systems can offer promising strategies for the intrinsic limitations of essential oils, improving their stability, controlled release, and skin compatibility. By encapsulating them into nanoscale structures, irritation can be reduced, while bioavailability can be enhanced.

Maryam Fakhariha et al. (2025) [8] examined the nanoencapsulation of essential oils from *Thymus vulgaris* (thyme) and *Salvia officinalis* (sage) using hollow silica nanospheres and hollow polymer nanocapsules. Essential oils are limited by volatility and degradation, resulting in reduced stability and functional performance. In this study, hollow silica nanospheres were synthesized via a sol–gel method with tetraethyl orthosilicate, and UF-hollow polymer nanocapsules were produced through in situ polymerization to encapsulate the oils. Morphological and physicochemical properties were assessed using Fourier emission infrared spectroscopy, field emission scanning electron microscopy (SEM), and dynamic light scattering (DLS). The encapsulated formulations exhibited high structural integrity, uniform particle size distribution, and enhanced oil loading. Over a 102-day period, the nanoencapsulated systems demonstrated controlled-release profiles and improved stability relative to non-encapsulated oils. Antimicrobial assays against *Escherichia coli* and *Staphylococcus aureus* showed that thyme oil encapsulated in hollow silica nanospheres and retained strong antibacterial activity at lower concentrations than when loaded in hollow polymer nanocapsules, indicating increased stability and sustained bioactivity through nanoencapsulation.

Perla Giovanna Silva-Flores et al. (2023) [9] assessed polymeric nanocapsules loaded with essential oils from *Rosmarinus officinalis* (rosemary) and *Lavandula dentata* (lavender) as skin delivery systems, with emphasis on stability, antioxidant activity, and dermatokinetic properties. The hydrophobic and volatile nature of essential oils often results in rapid evaporation and chemical degradation, making their use more difficult in topical applications. In this study, the oils were characterized and incorporated into polymeric nanocapsules via nanoprecipitation, producing stable nanosized carriers that increased skin surface residence time. Antioxidant activity, measured by the ferric thiocyanate method showed that nanoencapsulated oils maintained high antioxidant efficacy after formulation. Ex vivo studies using porcine skin in modified Franz diffusion cells, and tape-stripping techniques demonstrated greater deposition of key oil components in the stratum corneum when encapsulated, compared to free oils.

Essential oils are mixtures of volatile, bioactive compounds that have been studied for antimicrobial, antioxidant, and anti-inflammatory effects. Their natural origin and broad biological activity support their use in pharmaceuticals, cosmetics, food preservation, and biomedical materials. However, essential oils have several limitations, such as high volatility, low solubility in water, chemical instability due to oxidation and photodegradation, and uncontrolled release. These factors reduce their effectiveness and reproducibility.

Nanoformulation methods, especially core–shell nanostructures, have been developed to address these problems. Encapsulating essential oils in a protective shell can improve chemical stability, increase dispersibility in water, allow for controlled or responsive release, and lower toxicity by reducing the required dose. Recent research has focused on eco-friendly nanoformulations, using biodegradable, biocompatible, and renewable materials like polysaccharides, proteins, lipids, and green-synthesized polymers. Low-impact fabrication methods are also being prioritized [10].

Although there have been many advances in this area, the literature is still fragmented. There are differences in terminology, standards for characterization, and limited comparison of environmental aspects. A focused review of eco-friendly core–shell EO nanoformulations is needed to evaluate material choices, fabrication methods, functional performance, and sustainability. This can help identify design principles that support both effective technology and environmental responsibility.

**Figure 1:**
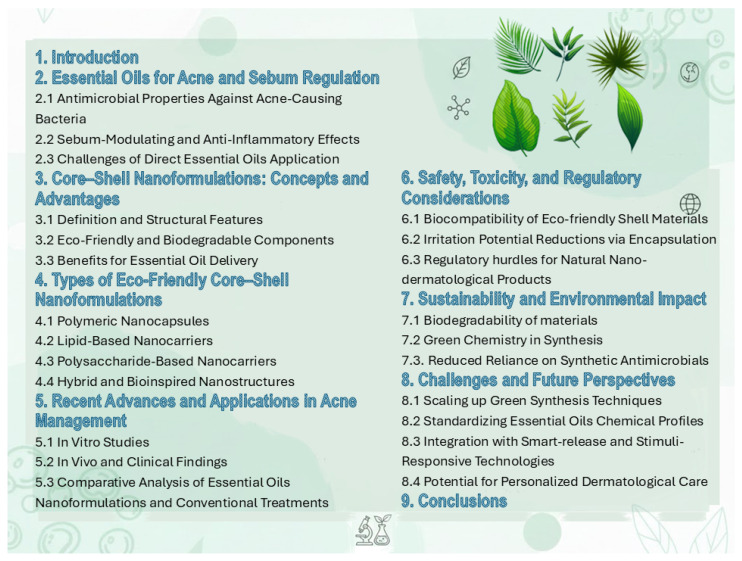
The framework of this review article.

This review examines recent progress in developing eco-friendly core–shell nanoformulations with essential oils. It focuses on material selection, formulation strategies, and functional performance. The discussion focuses on systems constructed from biodegradable, biocompatible, and renewable materials, showing how sustainable design principles are used in advanced nanoencapsulation technologies. Moreover, the review analyzes natural essential oils as active cores, describing their technological potential and formulation challenges. It also gives an overview of environmentally friendly shell materials, such as polysaccharides, proteins, lipids, and safe synthetic polymers. Main fabrication methods are assessed for environmental impact, scalability, and ability to produce well-defined core–shell structures. Finally, this review also examines physicochemical and functional characterization, focusing on stability, encapsulation efficiency, and controlled-release behaviour as important factors for practical use. Case studies from the food, pharmaceutical, biomedical, and cosmetic sectors are included to show how eco-friendly core–shell systems can improve the performance and safety of essential oils (Figure 1). This review differs from previous works by focusing specifically on core–shell nanoformulations for essential oil delivery in acne treatment, including analyses of polymeric, lipid, polysaccharide-based and hybrid carriers. It integrated ex vivo and in vivo data to better examine penetration, retention, and release. This review also addresses safety, toxicity, and regulatory considerations, as well as standardization and integration with smart-release technologies. Combining these topics offers a broader perspective than earlier reviews.

## 2. Essential Oils for Acne and Sebum Regulation

### 2.1. Antimicrobial Properties Against Acne-Causing Bacteria

#### 2.1.1. *Cutibacterium acnes* and *Staphylococcus epidermidis*

*Cutibacterium acnes* and *Staphylococcus epidermidis* are key microbial agents involved in the inflammation and progression of acne, making them important therapeutic targets. Studies on essential oils of plant origin, such as *Citrus reticulata*, *Zanthoxylum schinifolium* and tea tree oil, demonstrate potent antibacterial activity against these microorganisms, often comparable to or superior to conventional antibiotics. This efficacy is mainly attributed to the synergistic action of terpene components, supporting the use of natural antimicrobials as alternative or complementary approaches to acne management.

Research conducted by He-Shuai Hou et al. (2019) [11] examined the hydrodistilled *Citrus reticulata Blanco* peel essential oil. The analysis focused on its antibacterial activity against *Cutibacterium acnes*. This bacterium is considered as the most important pathogenic species in acne development. After essential oil distillation, the product was chemically characterized by GC-MS. This assay revealed that the composition is dominated by terpenes, with d-limonene identified as the major constituent. The antimicrobial potential of the oil against *C. acne* was measured using the disc diffusion technique. Additional tests were performed on *Escherichia coli*, *Staphylococcus aureus*, and *Bacillus subtilis* to compare sensitivity across bacterial strains. The largest inhibition zone was recorded for *E. coli*, although *C. acne* also showed sensitivity to the essential oil. A subsequent part of the study compared the activity of the citrus essential oil with commonly prescribed acne antibiotics such as erythromycin, clindamycin, and tetracycline, using the disc diffusion method again. The results showed that the essential oil created a wider inhibition zone than any of the tested antibiotics.

Shenglan Liao et al. (2022) [12] investigated the antibacterial properties of *Zanthoxylum schinifolium Sieb. Et Zucc* essential oil. The research focused on its activity against *Staphylococcus epidermidis*. The authors suggest that the examined essential oil could become a well-tolerated and low-risk antibacterial agent. Previous research demonstrated its activity against several foodborne microbes and pathogenic fungi. In this study, the essential oil showed strong inhibition of *Staphylococcus epidermidis*. Minimum inhibitory concentration (MIC) was 2.5 mg/mL, and the minimum bactericidal concentration (MBC) was 5 mg/mL. These values indicated high antibacterial potential. The researchers also examined antibacterial behaviour of the main constituents—linalool, dihydrolinalol, β-ocimene, and nerolidol. Their MIC values ranged from 2.5 to 10 mg/mL. The remaining minor components exhibited weak effects. All major constituents individually had MBC values of 20 mg/mL or above. These findings confirmed that the synergistic interaction among multiple components of the oil is essential for effective strain inactivation.

Recent research conducted by Sultan Pekacar et al. (2025) [13] investigated the antimicrobial activities of various tea tree oil samples against a range of control strains. Among these, the key contributors of acne development were analyzed, which are *Staphylococcus epidermidis* and *Propionibacterium acnes*. Microdilution assay tested the antimicrobial activity of tea tree oil samples against different microorganisms. Gram-positive and Gram-negative bacteria, dermatophytes, and fungi were included in this analysis. The MIC and MBC values for Gram-positive and Gram-negative bacteria ranged from 0.5% to 6% (*v*/*v*). All samples demonstrated the strongest antimicrobial and anti-biofilm effects against *P. acnes* and *Lactobacillus plantarum*, with MIC and MBC values of 1% (*v*/*v*). For yeast-like fungi and dermatophytes, the examined essential oils showed anti-biofilm, antifungal, and anti-dermatophytic activities within the 1–4% (*v*/*v*) range. The organic tea tree oil sample demonstrated consistent effect across all tested strains. In the case of *S. epidermidis*, the MIC was 2% (*v*/*v*) and the MBC 6% (*v*/*v*).

#### 2.1.2. Evidence of Antibacterial, Anti-Inflammatory, and Antioxidant Effects

Numerous studies have documented that essential oils exhibit combined antibacterial, anti-inflammatory, and antioxidant activity, properties critical for the management of acne. Citrus oils, thyme, oregano, clary sage and tea tree oil effectively inhibit inflammatory mediators, such as nitric oxide, while also exhibiting potent activity against *Cutibacterium acnes* and *Staphylococcus epidermidis*. The results are mainly attributed to the synergistic action of monoterpenes, phenolic and terpenic compounds, with low cytotoxicity, supporting their use as multifunctional natural therapeutic agents.

**Figure 2:**
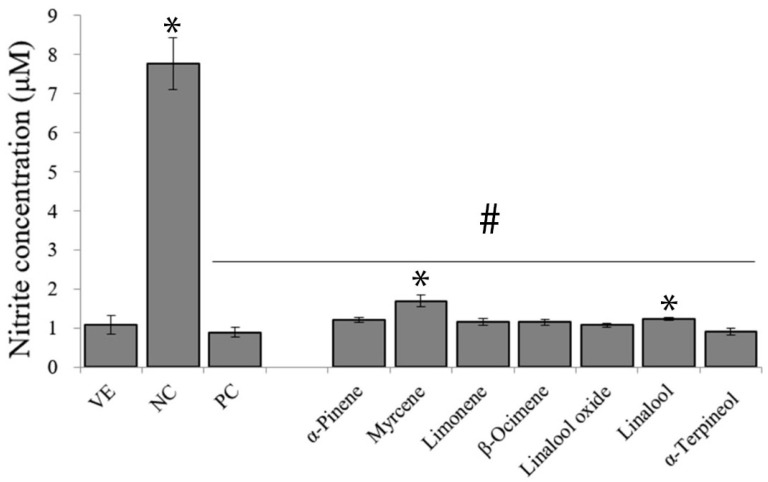
Inhibitory effects of single compounds on nitric oxide production. Data are presented as mean ± standard deviations. * *p* < 0.05 compared with untreated cells (VE); # *p* < 0.05 compared to lipopolysaccharide-treated cells (NC), dexamethasone (PC) [14].

Jiyoon Yang et al. (2023) [14] examined the anti-inflammatory potential of essential oils derived from 21 different citrus cultivars. Results of this study show that citrus essential oils inhibit nitric oxide production in LPS-stimulated macrophages. The oils also minimized the release of other inflammatory mediators due to lowering nitric oxide levels. These findings clearly demonstrate anti-inflammatory potential of examined oils. It was found that monoterpenes play an essential role in this effect because they can decrease inflammation and influence main chemical mediators involved in the process. *C. japonica* oil showed the strongest anti-inflammatory effect. Even though α-terpineol is linked with this activity, that oil contained less of this compound than the other samples. This finding suggests that the activity of citrus essential oils is the combined action of many constituents that produces the strongest effect and does not depend on a single one (Figure 2).

The properties of essential oils obtained from oregano, thyme, and sage were investigated by Erasmia Sidiropoulou et al. (2022) [15]. This research included characteristics of antioxidant, antimicrobial, anti-coccidal, and anti-inflammatory actions. The antimicrobial potential of oregano essential oil is related to its high levels of carvacrol, thymol, y-terpinene, and p-cymene. Thyme essential oil also contains a variety of aromatic compounds, for example, thymol and carvacrol. Those ingredients have a role in antioxidant and antimicrobial effects. The third examined oil, derived from *Salvia fruticosa* (sage), is rich in phenolic and terpenoid constituents, mainly eucalyptol and camphor. The in vitro results demonstrated that every essential oil could inhibit bacterial growth and prevent parasite invasion. Among them, oregano and thyme oils showed the strongest antioxidant activity. The highest anti-inflammatory effect displayed thyme essential oil, and the most important part was that the oils also exhibited very low cytotoxicity (Table 1).

Research conducted by Farah M. Abdelhamed et al. (2022) [16] examined five frequently used essential oils. The researchers explored its antimicrobial potential, an important factor managing acne vulgaris. Microbiological assays showed that essential oils acquired from thyme, tea tree, and clove were effective in inhibiting the growth of *Cutibacterium acnes* and *Staphylococcus epidermidis*. The most rapid and potent bactericidal activity was demonstrated by thyme essential oil. Complete elimination of the initial bacterial load appeared after 10 h for *Cutibacterium acnes* and 6 h for *S. epidermidis*. The strongest anti-biofilm action was observed in thyme essential oil. These findings prove that thyme essential oil is a promising antibacterial agent derived from plants.

### 2.2. Sebum-Modulating and Anti-Inflammatory Effects

Sebum overproduction in acne is often linked to inflammatory signalling and oxidative stress. Essential oils have proven to be promising bioactives capable of modulating inflammatory mediators and redox balance, so they can target multiple pathways implicated in acne pathogenesis. The following studies highlight key molecular and cellular mechanisms underlying the sebum-modulating and anti-inflammatory effects of essential oils.

A study by Jiyoon Yang et al. (2023) [14] examined the anti-inflammatory activity of citrus essential oils. Inflammation is a natural defence mechanism induced by macrophages. Investigation of essential oils’ activity involved measuring levels of key inflammatory markers, including nitric oxide, tumour necrosis factor-α, cyclooxygenase-2, inducible nitric oxide synthase, interleukin-1β, and interleukin-6. Macrophages produce pro-inflammatory cytokines and enzymes upon exposure to lipopolysaccharide. Such enzymes are inducible nitric oxide synthase or cyclooxygenase-2. This process led to the formation of mediators like nitric oxide and prostaglandin E_2_, with the first helping to eliminate pathogens, while its excessive production increases leakiness of blood vessels. Other nitric oxide overproduction results include swelling or intensifying inflammation. This highlights the fact that effective suppression of inflammatory responses requires reduced levels of mediators like nitric oxide, prostaglandin E_2_, and related cytokines.

Moreover, in a study by Farah M. Abdelhamed et al. (2022) [16], the mechanism of antimicrobial activity of several essential oils was evaluated. The major focus was on thyme essential oil. The study suggested that the antimicrobial action of thyme oil came from its ability to disrupt the bacterial cell membrane. As a result, intracellular components leaked from the cells. Transmission electron microscopy (TEM) imaging confirmed visible morphological damage in acne-causing bacteria after exposure to the essential oil. That kind of reaction was observed on *Cutibacterium acnes* and *Staphylococcus epidermidis* at the essential oil’s MBC. Chemical profiling using GC-MS identified thymol as the dominant phenolic constituent, suggesting that it is responsible for the thymol essential oil’s biological activity.

Song-Xue Yang et al. (2026) [17] reported that essential oil from *Saposhnikovia divaricata* (EOSD) strongly suppresses *Cutibacterium acnes*-induced activation of the NOD-Like Receptor Pyrin 3 inflammasome. The study also clarified the molecular mechanisms involved. In vitro and in vivo experiments showed that EOSD acts on several inflammatory and oxidative pathways related to acne development. EOSD reduced NOD-Like Receptor Pyrin 3 inflammasome activation, which plays a key role in innate immune responses. Importantly, this effect was observed after stimulation with different triggers, including adenosine triphosphate, nigericin, and *Cutibacterium acnes*. This suggests a broad anti-inflammatory activity of EOSD. In addition, EOSD protected mitochondrial structure during inflammatory stress. Electron microscopy revealed that *Cutibacterium acnes* caused mitochondrial swelling, cristae damage, and membrane rupture. These alterations were clearly reduced after EOSD treatment.

Omprakash Mohanta et al. (2025) [18] investigated the anti-inflammatory effects of essential oil from *Leonotis nepetaefolia* (LNLEO). The oil lowered the expression of pro-inflammatory cytokines, including tumour necrosis factor-α, interleukin-1β, and interleukin-6. It also reduced the levels of inflammatory enzymes such as inducible nitric oxide synthase and cyclooxygenase-2. In addition, LNLEO decreased intracellular ROS production. LNLEO also increased the activity of endogenous antioxidant enzymes, including endogenous antioxidant enzymes (SOD, GSH, Gpx, and CAT). The essential oil reduced oxidative stress by decreasing Kelch-like ECH-associated protein 1 expression and increasing nuclear factor erythroid 2-related factor 2 and heme oxygenase-1 levels. These effects were observed in a concentration-dependent manner. These results indicate that LNLEO can influence important pathways involved in inflammation and oxidative stress. Table 2 tabulates the biological activities of essential oils applied in acne treatment.

### 2.3. Challenges of Direct Essential Oils Application

Despite their promising biological activities and properties, the direct application of essential oils can be really challenging. High volatility, chemical instability, and dose-dependent cytotoxicity can compromise therapeutic efficacy and raise concerns regarding skin irritation and cellular damage. The studies below analyze these limitations and underscore the excessive need for more advanced delivery strategies to ensure safety and stability.

A. Prashar et al. (2004) [19] examined the cytotoxic effects of lavender essential oil (*Lavandula angustifolia*) and its principal constituents, linalool and linalyl acetate, on human skin cells. Although lavender oil is regarded as a mild aromatic oil with therapeutic uses, exposure at 0.25% (*v*/*v*) resulted in significant cytotoxicity in cultured human endothelial cells and fibroblasts, indicating potential for skin irritation and cellular damage upon direct topical application. Linalool demonstrated cytotoxicity similar to the complete oil, while linalyl acetate was even more cytotoxic, suggesting variability in the biological effects of individual constituents. The study attributed these effects to membrane damage and emphasized that even commonly used essential oils may present safety risks at relatively low concentrations when applied undiluted, presenting a major obstacle for unformulated essential oil use.

Eugenia Ganosi et al. (2023) [7] conducted a comprehensive stability analysis of essential oils from *Mentha × piperita*, *Mentha spicata*, *Origanum vulgare*, and *Thymus vulgaris*, focusing on the impact of thermal and storage conditions on chemical composition. Using GC-MS, the study monitored the essential oil profiles over 6 months in various containers at temperatures ranging from −20 °C to 45 °C. Major constituents remained relatively stable under low-oxygen conditions, but minor volatile components (<1%) degraded at higher temperatures and with light exposure, indicating oils were susceptible to oxidative and thermal instability. These results demonstrate that essential oils are inherently volatile and can undergo compositional changes even during short- to mid-term storage, making their direct use difficult in formulations that require consistent bioactive profiles.

Guilin Ren et al. (2022) [20] investigated the volatilization behaviour of volatile oils and mechanisms for controlling their release by solidifying them with porous starch, directly confronting the issues related to high volatility and poor stability in essential oil applications. Physical adsorption onto porous starch matrices enabled the conversion of tangerine peel volatile oils into solid powders and tablets, which were subsequently analyzed using GC-MS, X-ray diffraction (XRD), Thermogravimetric Analysis (TGA), and differential scanning calorimetry (DSC). The unformulated volatile oils exhibited a rapid loss of low-molecular-weight components upon air exposure, confirming that high vapour pressure and chemical composition are key factors driving the rapid loss of active constituents at room temperature. In contrast, incorporation into porous carriers slowed the release profile, demonstrating a viable strategy to mitigate volatility-related challenges and support consistent therapeutic efficacy and controlled dosing in topical and functional formulations.

A recent study by Xinhui Peng et al. (2025) [21] provided quantitative insights into the volatility profiles of essential oil constituents using an integrated multiscale analytical approach merging thermal analysis, GC, and molecular dynamics simulations. The study revealed that volatility is governed by molecular structure, weight, and functional groups, with lighter monoterpenes exhibiting rapid volatilization (e.g., >95% loss within two hours) compared to larger, less volatile compounds. The researchers also evaluated the modulation of volatilization using chemical fixatives and zeolite encapsulation, demonstrating that interactions between EO molecules and carrier surfaces can either delay or accelerate release depending on pore size and adsorption energy.

Taken together, the presented studies demonstrate that direct application of essential oils is challenged by a cytotoxic effect at relatively low concentrations, instability under thermal and light exposure, and rapid loss of volatile constituents. Experimental data confirm that both whole oils and individual components may undergo compositional changes during storage, which can affect their biological activity.

## 3. Core–Shell Nanoformulations: Concepts and Advantages

### 3.1. Definition and Structural Features

Core–shell nanostructures consist of a functional core surrounded by a protective shell, allowing the separation of structural and bioactive roles within the same nanosystem. This architecture improves the stability, solubility, and bioavailability of hydrophobic compounds, such as essential oils, while also allowing for controlled release and enhanced biocompatibility. Natural polymers and lipid materials are often used as shells, imparting mechanical integrity and functional flexibility to nanosystems.

Functional performance in nanoformulation systems is closely linked to structural parameters. The loading capacity is determined mainly by the core volume, which increases disproportionately with the core radius. As a result, even small increases in particle size can significantly enhance the maximum loading capacity when solubility is unchanged. In contrast, increasing the shell mass without expanding the core leads to a reduction in relative loading per total particle mass. Encapsulation efficiency depends on the surface-to-volume ratio: smaller particles have a greater interfacial area relative to their volume, which facilitates diffusional loss of the encapsulated compounds during fabrication. Larger particles, by comparison, improve retention and thus increase encapsulation efficiency. Release kinetics are governed by shell thickness and matrix density. A thicker shell increases the diffusion path length and slows release, while higher crosslink density or greater structural compactness further restrict molecular mobility and decrease the release rate, often in a non-linear fashion. Burst release is more pronounced in smaller particles, as a larger proportion of the active compound is located near the surface. In lipid-based systems, higher matrix crystallinity also restricts diffusion and extends the release period. These quantitative structure–function relationships provide a systematic basis for optimizing nanoformulation design to achieve targeted loading efficiency, retention, and controlled dermal delivery.

Dheebika Natrajan et al. (2015) [22] investigated the formulation of chitosan–alginate nanocapsules for encapsulating hydrophobic essential oils, specifically turmeric and lemongrass oils, which are known for their antibacterial, antifungal, antioxidant, and anticarcinogenic activities. Direct application of these oils is limited by their volatility, instability, and poor water solubility. In this study, alginate (AL) and chitosan (CS), two naturally occurring biopolymers with biodegradable and biocompatible properties, were selected as core–shell materials to form stable nanocapsules via ionic gelation. Physicochemical characterization using SEM, Fourier transform infrared (FTIR) spectroscopy, and ultraviolet–visible (UV-Vis) spectroscopy confirmed that optimized concentrations (0.3 mg/mL AL and 0.6 mg/mL CS) yielded spherical particles with sizes below 300 nm and good colloidal stability. The essential oil-loaded nanocapsules exhibited significantly enhanced antiproliferative activity against A549 cancer cell lines compared with non-encapsulated oils, suggesting improved bioavailability and functional performance.

**Figure 3:**
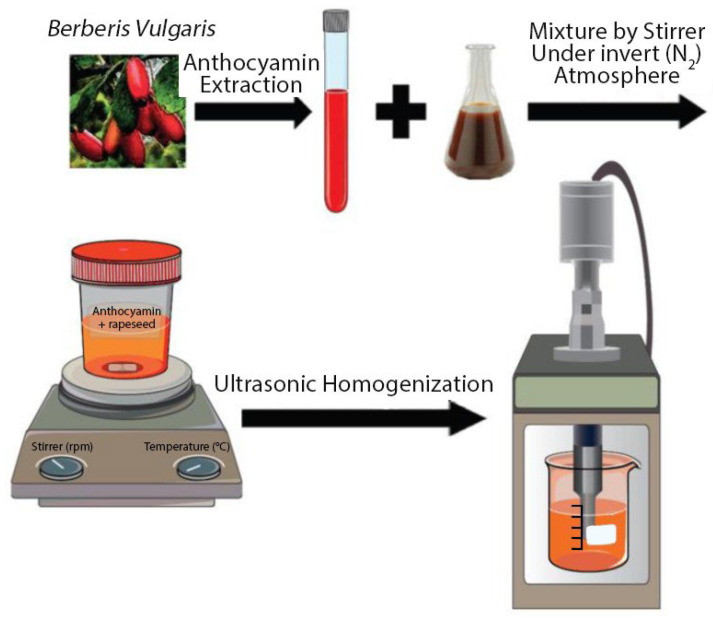
Schematic illustration of preparation of anthocyanin compounds (AC) incorporated in rapeseed nanoliposome [23].

Mina Homayoonfal et al. (2021) [23] developed lipid nanoliposomes based on rapeseed lecithin to encapsulate anthocyanin compounds and systematically examined how extract concentration influenced their physicochemical properties. The nanocarriers were fabricated using a hydration and ultrasonication technique, yielding stable nanoparticles (NPs) with mean diameters of 141–196 nm, a negative surface charge, and narrow size distributions. Higher anthocyanin loading resulted in increased particle size, polydispersity index, surface charge, and encapsulation efficiency, reaching a maximum encapsulation efficiency of 43% at 9% extract content. Structural analyses indicated reduced membrane fluidity due to hydrophobic interactions between anthocyanins and lipid chains, while TEM revealed predominantly spherical vesicles with improved morphology following extract incorporation. Cytocompatibility studies using human mesenchymal and fibroblast cells confirmed the nanoliposomes’ non-toxicity, supporting the use of rapeseed lecithin as a biodegradable, biocompatible lipid material suitable for sustainable nanoformulation platforms (Figure 3).

Mohamed S. Hasanin et al. (2025) [24] examined chitosan-based nanoemulsions loaded with lemon peel, turmeric, and black seed essential oils, highlighting the role of chitosan as a biopolymer shell material that enhances stability and biological efficacy. Using nanochitosan (NCh) as the structural shell component, the researchers formulated emulsions in which the hydrophobic essential oil core was encapsulated within a chitosan-stabilized nanoscale matrix. Physicochemical characterization using FTIR, DLS, and high-resolution TEM confirmed homogeneous particle size distributions and stable core–shell morphology at the nanoscale (~121 nm). When tested against fungal strains associated with mucormycosis, the NCh-loaded essential oil nanoemulsions exhibited pronounced antifungal activity—producing inhibition zones between 17 mm and 23 mm—that outperformed several commercial synthetic antifungal agents [24].

The cited studies illustrate the core–shell nanoformulation concept as a strategy for structural compartmentalization, in which a bioactive core (e.g., essential oils or anthocyanins) is protected and stabilized by a biocompatible shell, such as chitosan, alginate, or lecithin. The shell governs colloidal stability, surface charge, and biological interactions, while the core retains functional activity. Across these examples, the architecture enhances stability, solubility, and bioavailability, leading to improved antiproliferative or antifungal efficacy compared with non-encapsulated compounds.

### 3.2. Eco-Friendly and Biodegradable Components

#### 3.2.1. Natural Polymers

Natural polymers, such as chitosan, alginate and carboxymethylcellulose (CMC), are key building blocks of eco-friendly and biodegradable core–shell nanoformulations. Their biocompatibility and ability to form stable nanostructures and functionality through ionic or hydrogen bonding interactions make them ideal for the encapsulation and controlled release of sensitive essential oils. The following studies highlight the role of natural polymers as viable shells and matrices in advanced nanodelivery systems.

Yu-Hsuan How et al. (2024) [25] developed antibacterial films based on a composite of CMC and chitosan incorporated with a nanoemulsion of *Persicaria minor* essential oil for sustainable food packaging applications. The poor aqueous solubility and susceptibility to oxidation of essential oils challenge their direct use; this study addressed that by first formulating a stable nanoemulsion of the EO, then embedding it into a biodegradable CMC-CS film matrix. The authors evaluated the effects of different essential oil emulsion concentrations (0–12% *v*/*v*) on film properties, finding that nanoemulsified EO reduced droplet size and enhanced antibacterial activity against *Escherichia coli* and *Bacillus subtilis*. Films with higher EO content demonstrated improved antioxidant activity and more flexible mechanical properties, with optimized water solubility and surface morphology.

Ioanna Pitterou et al. (2024) [26] investigated the development of hybrid alginate hydrogels incorporating essential oils loaded in chitosan NPs, focusing on their structural properties and functional performance for potential biomedical applications. Resolving the issues of direct essential oil use—such as high volatility and poor aqueous compatibility—this study prepared a system in which chitosan (CS) served as a biopolymer matrix encapsulating lavender and *Mentha* essential oils, forming CS-NPs via ionic gelation in an acidic environment created by a natural deep eutectic solvent (NADES). The CS-NPs, with hydrodynamic diameters of approximately 130.7 nm and 143.4 nm, were embedded into a crosslinked alginate hydrogel network. Comprehensive characterization revealed a stable polymeric network with a high-water retention capacity (>80%) even under acidic conditions, indicating robust hydrogel integrity. Antimicrobial assays against Gram+ and Gram- pathogens demonstrated that the hybrid AL-CS system exhibited bioactivity comparable to conventional preservatives, illustrating how natural polysaccharide biopolymers, such as chitosan and alginate, can be strategically employed as eco-friendly shell and matrix materials to stabilize and deliver volatile essential oils within biodegradable core–shell nanoformulations.

**Figure 4:**
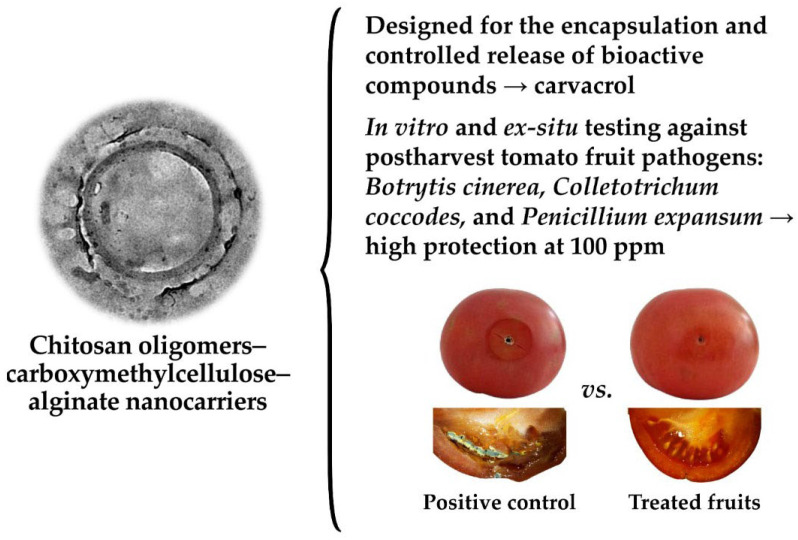
Schematic representation of the study by Eva Sánchez-Hernández et al. [27].

In the study by Eva Sánchez-Hernández et al. (2024) [27], the authors explored the encapsulation of carvacrol, a phenolic compound abundant in many essential oils, within nanocarriers assembled from a ternary blend of chitosan oligomers (COS), alginate (ALG), and CMC for postharvest tomato protection. Essential oils and their bioactive constituents, such as carvacrol, are highly volatile and prone to degradation, which limits their direct application in food and agricultural systems. To reduce these difficulties, the research team synthesized hollow nanospheres by complexing the three biopolymers with methacrylic anhydride and sodium tripolyphosphate as crosslinkers, yielding monodispersed particles with an average diameter of approximately 114 nm. TEM and spectroscopic analyses confirmed the formation of stable polymeric nanocarriers capable of efficiently encapsulating carvacrol with a loading capacity of about 20% and high encapsulation efficiency (85–89%). Functional testing against phytopathogenic fungi (*Botrytis cinerea*, *Penicillium expansum*, and *Colletotrichum coccodes*) showed that the carvacrol-loaded nanocarriers achieved MIC as low as 23 μg·mL^−1^, significantly lower than non-encapsulated carvacrol, demonstrating enhanced antifungal activity (Figure 4).

These studies show that natural polymers, such as chitosan, alginate, and CMC, serve as the basis for biodegradable core–shell nanoformulations, acting as protective shells or matrices for volatile essential oils. Through ionic and hydrogen-bond interactions, they create stable nanostructures that improve solubility and protect sensitive compounds from degradation. The resulting systems demonstrate enhanced stability, high encapsulation efficiency, improved mechanical or hydrogel properties, and significantly stronger antimicrobial or antifungal activity compared with non-encapsulated compounds, while maintaining eco-friendly and biocompatible characteristics suitable for food and biomedical applications.

#### 3.2.2. Green Synthesis Approaches

Green synthesis approaches utilize essential oils and phytochemicals as natural reducing and stabilizing agents to produce metal NPs, eliminating the use of toxic chemical reagents. These methods allow the creation of nanostructures with controlled morphological and functional characteristics, combining antimicrobial and antioxidant activity with a reduced environmental footprint. The following studies highlight the potential of green synthesis as a viable strategy for biomedical and dermatological applications.

Mir-Hassan Moosavy et al. (2023) [28] investigated the green synthesis of gold (AuNPs) and silver NPs (AgNPs) using *Mentha spicata* (spearmint) essential oil as both the reducing and stabilizing agent, eliminating the need for traditional toxic chemical reductants. In this study, aqueous solutions of chloroauric acid (HAuCl_4_) and silver nitrate (AgNO_3_) were mixed separately with the essential oil and incubated at room temperature for 24 h. Characterization by UV–Vis spectroscopy, DLS, TEM, SEM, FTIR spectroscopy, and XRD confirmed the formation of spherical AuNPs and AgNPs with well-defined crystalline structures. GC-MS analysis of the *M. spicata* oil identified phenolic and monoterpene components that facilitated the reduction in metal ions and acted as capping agents, providing colloidal stability. The green-synthesized NPs exhibited significant antibacterial, antioxidant, and cytotoxic activities, with AgNPs showing superior antimicrobial effects.

Vidya Vilas et al. (2016) [29] reported the green synthesis of silver nanocrystals using essential oil extracted from the leaves of *Coleus aromaticus* as a bioreductant and stabilizer, producing environmentally benign silver NPs. Under physiological pH and elevated temperature conditions (373 K), the essential oil reduced Ag^+^ ions to zero-valent silver, forming NPs characterized by UV-Vis spectroscopy, FTIR, TEM, and XRD, which revealed crystalline face-centred cubic nanospheroids and occasional nanorods with average dimensions around 26–28 nm. The phytochemical compounds contained in the essential oil, especially terpenes and phenol derivatives, actively contribute to the reduction and encapsulation of NPs, ensure their stability, and enable effective catalytic degradation of hazardous organic dyes in the aquatic environment.

Shahram Ahmadi et al. (2021) [30] performed a green synthesis of iron NPs (FeNPs) employing *Satureja hortensis* essential oil as a natural reducing and stabilizing agent. The study synthesized FeNPs with cubic morphology and particle sizes ranging from ~9.3 nm to 27 nm, as confirmed by UV-Vis spectroscopy, FTIR, XRD, and field emission SEM. The biologically synthesized NPs exhibited enhanced antimicrobial performance against selected Gram-positive and Gram-negative bacteria and the fungus *Candida albicans* compared to the essential oil alone. Additionally, the FeNPs showed notable anticancer activity against human cancer cell lines (K-562 and MCF-7) in 3-(4,5-dimethylthiazol-2-yl)-2,5-diphenyltetrazolium bromide (MTT) cytotoxicity assays.

### 3.3. Benefits for Essential Oil Delivery

#### 3.3.1. Enhanced Stability and Sustained Release

Nanoencapsulation is an effective strategy to enhance physicochemical stability and controlled release of essential oils, addressing their inherent volatility and susceptibility to degradation. Nanoformulations can protect bioactive compounds from thermal and oxidative stress, while enabling sustained and predictable release properties, by tailoring the carrier’s composition and structural architectures.

Mingcheng Zhang et al. (2024) [31] developed chitosan-based nanocapsules loaded with cumin essential oil. Nanoformulation was produced using the ionic gelation technique. The researchers examined the antibacterial potential of the nanocapsules against *Escherichia coli* and *Listeria monocytogenes*. Particular attention was on the controlled release profile of the encapsulated essential oil. Nanoencapsulation shields the cumin essential oil from volatilization and oxidative degradation during heating. This has been confirmed by DSC. This examination revealed that incorporating cumin essential oil into the chitosan matrix improved its thermal stability. The formulated nanocapsules exhibited inhibitory effects on both examined bacterial strains, with the prolonged and gradual release of the active compound. UV-vis absorption spectroscopy analyses confirmed that encapsulation promotes sustained-release behaviour of cumin essential oil.

In recent research, Junsi Yang et al. (2025) [32] explored the development of hollow solid lipid micro-NPs loaded with peppermint essential oil, employing a green atomization process based on CO_2_-expanded lipid mixtures. The study confirmed the effectiveness of this technique and demonstrated that the resulting particles provide controlled release and pronounced antibacterial activity. Although the formulations shared identical initial essential oil concentrations and comparable loading efficiencies, the composition of the lipid shell primarily dictated the release behaviour. Particles produced using fully hydrogenated soybean oil (FHSO) or soybean monoacylglycerols released the encapsulated oil more rapidly than other variants. In contrast, particles obtained from blends of FHSO and liquid oils released the essential oil slower, likely due to the higher viscosity of the lipid mixture and the resulting stronger shell.

Aneela Manzoor et al. (2023) [33] investigated strategies to improve the stability and functional efficiency of lavender, basil, and clove essential oils in the presence of pathogenic microorganisms. Their study compared the antibacterial performance of the oils before and after nanoencapsulation within microemulsion systems. Two pathogenic strains were used as experimental models. The antimicrobial response toward *Staphyloccocus aureus* and *Escherichia coli* was enhanced by a reduction in droplet size. Moreover, the encapsulated formulations demonstrated improved radical-scavenging activity against foodborne microbes in comparison with pure essential oils. The findings indicated that the surface characteristics, hydrophilicity, and biological activity of essential oils can be improved by nanoscale encapsulation.

#### 3.3.2. Improved Skin Penetration and Reduced Irritation

The efficiency of nanoformulations in acne therapy is strongly influenced by the layered organization of the skin and the presence of pilosebaceous units. The stratum corneum limits passive diffusion whereas hair follicles and associated sebaceous glands provide preferential pathways for particle penetration and retention. Ex vivo studies using human or porcine skin models show increased follicular accumulation of lipid-based and polumeric NPs compared with conventional formulations. Nano-formulated essential oil formulations have been shown to significantly improve the dermal penetration of active ingredients while reducing the irritation that often accompanies their application in free form. Through the reduction in droplet size and controlled release, systems such as nanoemulgels enhance transdermal transport and skin compatibility. The following studies demonstrate the advantages of these systems in terms of efficacy and safety in topical applications.

Recent work in the field of essential oil nanoformulations includes the study by Mahmoud Omidi et al. (2025) [34], who aimed to develop a lavender essential oil nanoemulsion gel for skin-lightening purposes. The researchers examined the cumulative penetration of lavender essential oil components—especially linalool—through rat skin, comparing the nanoemulsion gel with a standard lavender essential oil base gel. Their results showed that the nanoemulsion gel achieved higher penetration into and across the skin layers, indicating better suitability for transdermal delivery than the conventional gel. The nanoemulsion also exhibited lower cytotoxicity than pure lavender essential oil. Moreover, the developed gel produced no discomfort or skin irritation, demonstrated good epidermal compatibility, and promoted better user adherence.

Another recent study illustrating the dermatological potential of essential oil nanoformulations is the work of Hayder Kadhim Drais (2024) [35], which focused on the formulation and assessment of an antimicrobial skin-sanitizing nanoemulgel containing essential oils. Skin irritation test was performed on 30 volunteers using various essential oil nanoemulgel hand formulations. The results showed that none of the tested gels caused itching, irritation, or any unpleasant sensations, indicated good skin tolerability. The study also emphasizes the importance of in vitro antimicrobial testing in nanoemulgel development, as such evaluations are essential for determining a formulation’s effectiveness against pathogenic microorganisms.

Reham Mokhtar Aman et al. (2020) [36] explored the creation of nanostructured formulations designed to provide a regulated release of clove essential oil. An ex vivo experiment was performed on Wistar albino rat skin. The authors demonstrated that the clove essential oil nanoemulsion-based nanoemulgel provided the most effective permeation through the skin. The study also included nanoemulsion-based nanofibres and pure clove essential oil, which gave worse results. This superior performance aligns with the well-stablished advantages of nanoemulgels. This type of formulation enhances dermal permeation due to the characteristics of the polymeric gel matrix, including improved adhesion and penetration-enhancing properties. Histopathological assessments further confirmed normal skin integrity in mice treated with the nanoemulgel and nanofibre systems, indicating a positive skin safety profile.

## 4. Types of Eco-Friendly Core–Shell Nanoformulations

### 4.1. Polymeric Nanocapsules

Polymeric nanocapsules are a versatile and sustainable class of nanocarriers for the encapsulation of essential oils, offering protection from volatility and degradation, as well as controlled release. Biodegradable polymers such as poly(ε-caprolactone) (PCL) and chitosan form stable core–shell structures with high skin compatibility. The following studies highlight the role of polymeric nanocapsules in improving the stability, bioavailability and topical efficacy of essential oils.

Giuseppe Granata et al. (2018) [37] investigated the development of polymeric nanocapsules composed of biodegradable polyester PCL for the encapsulation of *Thymus capitatus* and *Origanum vulgare* essential oils. The nanocapsules were produced via nanoprecipitation, yielding well-defined core–shell structures with particle sizes of 171 and 175 nm. The use of PCL provided a biodegradable, biocompatible shell that stabilized the volatile essential oil components.

**Figure 5:**
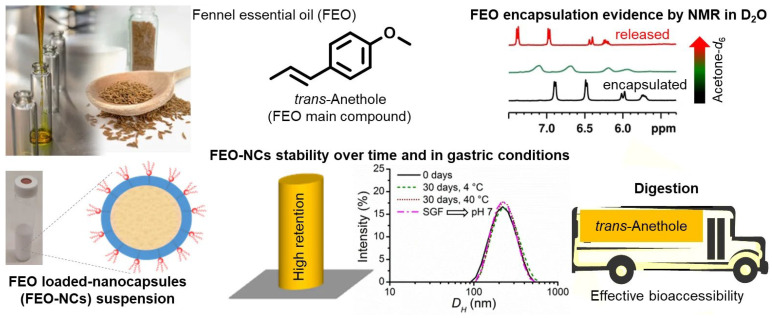
Schematic representation of the study by Granata et al. [38].

In another study, Granata et al. (2022) [38] reported the formulation of essential oil-loaded polymeric nanocapsules using biodegradable PCL as the shell material and Foeniculum vulgare essential oil as the lipophilic core. Nanocapsules were prepared through interfacial polymer deposition, resulting in spherical particles with a narrow size distribution and high colloidal stability. PCL’s biodegradability contributed to the formulation’s suitability for topical and biomedical applications. The authors emphasized that the polymeric shell enhanced essential oil retention and stability while preserving compatibility with biological environments, supporting the use of biodegradable polymers in eco-friendly nanocarrier design (Figure 5).

Moreover, in a study by Erveton P. Pinto et al. (2023) [39], copaiba (*Copaifera officinalis*) essential oil was encapsulated in nanocapsules incorporated into a chitosan matrix to produce biodegradable topical systems. Chitosan, a polysaccharide obtained from chitin, served as both a stabilizing and biodegradable polymeric component. The nanocapsules exhibited nanoscale dimensions and good structural integrity, demonstrating that chitosan-based polymeric systems can effectively host nanocapsules loaded with essential oils. The authors highlighted the sustainability and biodegradability of chitosan as key advantages for skin-related applications, reinforcing its relevance in eco-friendly nanoformulation strategies.

In the work of Mingcheng Zhang et al. (2024) [31], chitosan nanocapsules encapsulating cumin essential oil were developed and evaluated with particular emphasis on release behaviour. The biodegradable polymer matrix enabled sustained release of the encapsulated oil, attributed to diffusion through the chitosan shell and gradual polymer relaxation in aqueous media. Encapsulation efficiency values indicated effective loading of the essential oil within the nanocarriers. The controlled-release profile prolonged antimicrobial activity, demonstrating that biodegradable polymeric nanocapsules can modulate essential oil release kinetics while maintaining bioactivity.

In research by Ricardo M. González-Reza et al. (2020) [40], thyme essential oil was encapsulated in polymeric nanocapsules using PCL and ethylcellulose as carriers, with poly(vinyl alcohol) and pluronic^®^ F-127 as stabilizers. The nanocapsules were below 500 nm and showed encapsulation efficiencies of 60–70%, the highest for ethylcellulose formulations due to stronger polymer–oil interactions. In vitro release studies indicated sustained, diffusion-controlled release over 48 h, highlighting how polymer selection can tune both oil retention and release kinetics. These results indicate that biopolymeric nanocapsules can efficiently encapsulate and deliver essential oils, supporting their use in eco-friendly topical formulations for antimicrobial activity and sebum control.

Also, in a study by Arvind Negi et al. (2022) [41], antibacterial essential oils were encapsulated in chitosan NPs to investigate encapsulation efficiency and release behaviour. The nanocarriers were prepared using ionic gelation and related nanoencapsulation techniques, exploiting chitosan’s amino groups for effective entrapment of hydrophobic and volatile oil components. Several formulations demonstrated high encapsulation efficiencies (up to ~75% for basil essential oil), confirming the suitability of chitosan as a biodegradable polymeric matrix. Reported release profiles for chitosan-based systems exhibited biphasic behaviour, with an initial burst followed by sustained release controlled by diffusion and polymer matrix relaxation. Such controlled release prolongs the availability of active compounds at the application site and reduces the demand for frequent reapplication (Table 3).

### 4.2. Lipid-Based Nanocarriers

Lipid-based nanocarriers, such as solid lipid NPs (SLN) and nanostructured lipid carriers (NLC), have emerged as promising platforms for topical application in skin diseases characterized by increased sebum production, including acne. Their lipid composition favours interaction with the stratum corneum and sebaceous follicles, promoting improved skin penetration, controlled release, and improved tolerability of lipophilic bioactive compounds. The following studies highlight the role of these systems in improving both the efficacy and safety of topical anti-acne treatments.

In the study by Saman Ahmad Nasrollahi et al. (2021) [42], the authors developed 0.1% adapalene-loaded NLC for targeted topical delivery in acne treatment, a condition directly associated with sebum-rich skin environments. Adapalene is a third-generation retinoid commonly used in acne therapy but limited by low percutaneous absorption and irritation. To address this, NLC were formulated via probe sonication to encapsulate adapalene within a lipid matrix. Characterization of the NLC revealed suitable particle size, colloidal stability, and high drug loading, with entrapment efficiencies and physicochemical properties optimized for dermal adherence. A pilot clinical evaluation in patients with mild-to-moderate acne presented substantial reductions in acne severity and lesion counts after 12 weeks of treatment, alongside improvements in porphyrin production within sebaceous follicles, indicating effective delivery and activity in sebum-rich skin regions.

In research by Kaisar Raza et al. (2013) [43], a formulation of isotretinoin-loaded SLN was developed using a formulation-by-design approach for topical acne treatment, addressing both drug targeting in sebum-rich skin and reduced cutaneous irritation. Isotretinoin is a potent retinoid with known side effects when administered orally or topically; encapsulating it in biodegradable SLN aims to enhance dermal targeting and reduce systemic exposure. The optimized SLN formulation exhibited nanometre-range sizes (~75 nm) and high entrapment efficiency (~89%), forming drug micro-reservoirs within the lipid matrix that facilitated improved deposition in skin layers in an in vivo mouse model. Evaluation showed improved anti-acne potential and tolerability in sebaceous regions of the skin, suggesting that SLN not only improve localization within sebum-rich environments but also increase patient compliance by mitigating irritation often associated with retinoid therapy.

**Figure 6:**
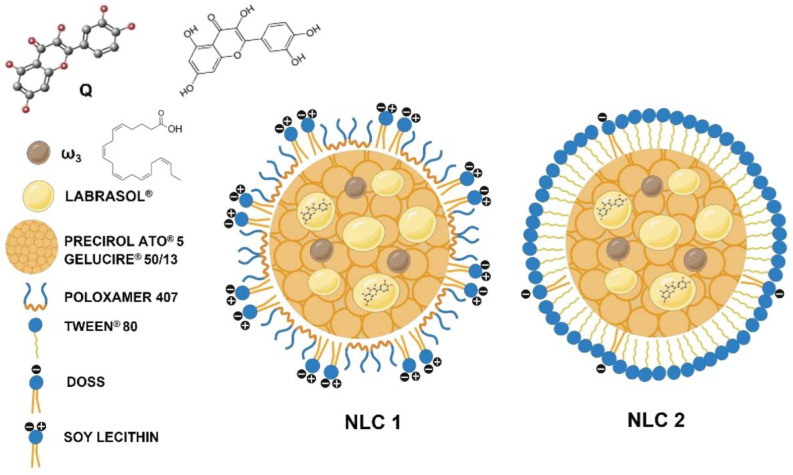
Schematic representation of nanostructured lipid carriers (NLCs) with or without quercetin (Q) and/or omega-3 fatty acid (ω3) [44].

In the work of Marlene Lúcio et al. (2023) [44], NLCs were developed and incorporated into enriched hydrogel systems to enable topical delivery of quercetin and omega-3 fatty acids, two bioactives with antioxidant and dermatoprotective potential. Even though it is focused on photoprotective and oxidative-stress-mitigating properties, this research is directly relevant to sebum-rich skin environments because it demonstrated that NLC can improve skin permeation and provide an occlusive effect, enhancing fluidization of the stratum corneum lipid matrix and facilitating dermal uptake of lipophilic actives. The optimized NLC (NLC2) exhibited physicochemical properties (size ~100–200 nm, stable zeta potential) that are within the optimal range for topical administration, a key criterion for treating oily skin conditions that require penetration through the sebaceous layers. The hydrogel vehicles further improved skin adhesion and prolonged the release of entrapped actives, supporting overall efficacy in cutaneous applications, where sebum and barrier properties influence delivery performance (Figure 6).

Hoda Atapour-Mashhad et al. (2024) [45] formulated coenzyme Q10-loaded SLN and NLC as potent antioxidant carriers for topical skin applications. Although the primary focus was on antioxidant and anti-tyrosinase effects, the study showed that both SLN and NLC systems exhibited high entrapment efficiency (>80%), physicochemical stability, and favourable in vitro release profiles, features essential for efficacy in sebum-rich skin environments where antioxidants must penetrate the lipid-rich barrier. The in vivo penetration studies also indicated that the carriers enhanced dermal uptake of coenzyme Q10 compared with conventional formulations, supporting lipid NPs’ ability to deliver lipophilic molecules effectively through the stratum corneum and sebaceous regions. The sustained-release behaviour observed suggests that these lipid carriers can prolong their active presence in oily skin conditions, providing enhanced bioavailability and potential benefits in acne-related oxidative stress contexts.

### 4.3. Polysaccharide-Based Nanocarriers

Polysaccharide nanocarriers, including systems based on alginate, chitosan, and pectin, are biodegradable, biocompatible, and ecologically sustainable platforms for topical pharmaceutical applications. Their ability to self-assemble into core–shell structures through electrostatic interactions facilitates the effective encapsulation and protection of bioactive molecules, while allowing for their controlled and sustained release. The following studies highlight the functional flexibility of polysaccharide nanocarriers in optimizing antimicrobial, anti-inflammatory and antioxidant activity in dermal systems.

In the study by H.T.P. Nguyen et al. (2016) [46], the authors focused on developing calcium alginate-based core–shell nanocarriers designed to enhance the topical delivery of hydrophobic active molecules such as curcumin, confronting the problem of poor aqueous solubility in dermatological and cosmetic formulations. The nanocarriers were prepared at room temperature, without organic solvents, using an accelerated nanoemulsification and polymer crosslinking method, yielding particles with a hydrodynamic diameter of approximately 200 nm and a negative surface charge (~−30 mV), properties favourable for skin application. Curcumin was encapsulated with high efficiency (~95%) within the alginate shell, and its antioxidant activity was fully retained. When applied to excised human skin, the curcumin-loaded alginate nanocarriers led to significant accumulation of the active molecules in the upper skin layers, demonstrating the capability of alginate core–shell structures to transport hydrophobic bioactives into skin-relevant strata effectively.

In research presented by Adam J. Friedman et al. (2013) [47], the authors developed chitosan–alginate core–shell NPs as biodegradable nanocarriers for targeting cutaneous pathogens. The NPs, approximately 50 nm in size, were produced by electrostatic complexation of cationic chitosan and anionic alginate, yielding stable polysaccharide-based structures. The system exhibited strong antimicrobial activity against *Propionibacterium acnes* and significantly reduced pro-inflammatory cytokine production in stimulated keratinocytes and monocytes, demonstrating both antimicrobial and anti-inflammatory effects. Furthermore, encapsulation of benzoyl peroxide within the NPs enhanced antibacterial activity while lowering cytotoxicity compared with the free drug.

In the work by Chiara Amante et al. (2022) [48], the authors developed alginate–pectin core–shell microparticles incorporating nanoemulsions loaded with curcumin, a model antimicrobial agent for wound-healing applications. Spray-drying was employed to produce in situ gelling powders composed of alginate and pectin, which rapidly formed hydrogels upon contact with simulated wound fluid. SEM revealed a transformation in particle morphology from irregular to spherical shapes with nanoemulsion incorporation. At the same time, rheological analysis indicated that the hydrogels exhibited elastic properties suitable for tissue contact. The core–shell composite gels sustained the release of curcumin-loaded nanoemulsions over 24 h. They demonstrated cytocompatibility with HaCaT human keratinocyte cell lines, confirming their potential as polysaccharide nanocomposites for prolonged topical delivery and support of wound healing.

In research by Thuy Thi Thanh Nguyen et al. (2023) [49], the authors fabricated pectin/chitosan composite films as biopolymer-based antibacterial systems incorporating biosynthesized silver NPs (AgNP) for potential wound-dressing applications, representing a core–shell-like composite structure at the film scale. The passion fruit peel pectin and chitosan matrix was combined with green-synthesized AgNP to enhance mechanical and antimicrobial performance. Characterization showed that increased AgNP concentration improved water content, water vapour permeability, and antibacterial activity against *Staphylococcus aureus*, *Bacillus cereus*, *Pseudomonas aeruginosa*, and *Klebsiella pneumoniae*. In vivo testing demonstrated complete wound closure within 15 days when applied to a wound model, highlighting that pectin/chitosan polysaccharide networks can function as biodegradable matrices that protect and deliver antimicrobial agents to the wound site.

### 4.4. Hybrid and Bioinspired Nanostructures

Hybrid and bioinspired nanostructures constitute an innovative class of sustainable nanomaterials, where biological derivatives are combined with inorganic nanoscales through environmentally friendly synthesis methods. The use of plant-derived phyto-chemicals as natural reducing agents and surface stabilization agents allows the production of metal and metal oxide NPs with increased biocompatibility and a limited ecological footprint. The following studies highlight the functional value of these bioinspired nanostructures in antimicrobial and antioxidant applications, as well as in dermatologically relevant biomedical uses.

In research by Anshu Saini et al. (2024) [50], the authors reported an environmentally benign strategy for synthesizing AgNPs using aqueous leaf extracts from *Azadirachta indica* (Neem), *Mentha piperita* (Mint), *Ocimum tenuiflorum* (Tulsi), and *Cynodon dactylon* (Bermuda grass). This method, rather than relying on conventional chemical reducing agents, exploited the phytochemicals present in the plant extracts to reduce Ag^+^ ions to metallic silver and stabilize the resulting NPs at the same time. The evolution of characteristic surface plasmon resonance peaks confirmed the biogenic synthesis in the 307–381 nm range, and the NPs demonstrated good colloidal stability. The green-synthesized AgNPs were successfully applied in catalytic reactions and showed promising effects on seed germination and plant vigour, bringing to light their eco-friendly synthesis and functional use across biological systems.

In a recent study by Farhat Gul et al. (2025) [51], Ag_2_O NPs were synthesized via an eco-friendly route using *Catharanthus roseus* leaf extract as a biological reducing and capping agent. The resulting NPs were characterized by SEM, FT-IR, and EDX to confirm size, morphology, and elemental composition. Antimicrobial assays demonstrated that the green-synthesized Ag_2_O NPs significantly inhibited both bacterial and fungal growth, with inhibition zones of ~19–21 mm in standard tests. The research emphasized the beneficial role of phytochemicals present in the plant extract in both reducing metal ions and stabilizing the formed NPs, facilitating antimicrobial efficacy without hazardous chemicals.

A study by Ali Mohammadi et al. (2025) [52] focused on the green synthesis of zinc oxide NPs (ZnO-NPs) using *Punica granatum* fruit peel extract as a natural reducing and stabilizing agent. Rather than employing hazardous chemical reagents typically used in nanoparticle fabrication, this study leveraged the high content of polyphenols, flavonoids, and tannins in the pomegranate peel extract to mediate the reduction of zinc precursors and capping of the resulting ZnO-NPs. The synthesized NPs exhibited a spherical morphology and homogeneous distribution, with a hydrodynamic size of ~187 nm and a negative surface charge indicative of colloidal stability, as confirmed by DLS, field emission FE-SEM, and XRD. Importantly, green-synthesized ZnO-NPs demonstrated significantly higher biocompatibility toward human foreskin fibroblast cells and lower haemolytic activity compared with chemically synthesized counterparts, as shown by MTT and haemolysis assays.

In a study by Bassant Naiel et al. (2022) [53], the authors adopted a green, sustainable protocol to synthesize ZnO NPs using the aqueous extract of *Limonium pruinosum*, a halophytic plant, as a natural reducing, capping, and stabilizing agent. UV-Vis spectroscopy, FT-IR, SEM, TEM, and XRD analyses confirmed the successful formation of ZnO nanostructures with an average size of ~41 nm exhibiting hexagonal/cubic crystallinity. The plant extract served a triple role in phytofabrication: reducing Zn^2+^ ions, providing surface capping to prevent uncontrolled aggregation, and stabilizing the final NPs. Biologically, the ZnO NPs demonstrated antimicrobial and antioxidant activities, as well as cytotoxicity against human skin cancer cells in a dose-dependent manner, while being biocompatible with normal human fibroblasts at safe concentrations. Table 4 tabulates the four types of eco-friendly core–shell nanoformulations in terms of raw material characteristics, encapsulation efficiency, release kinetics, skin biocompatibility and scalability.

## 5. Recent Advances and Applications in Acne Management

### 5.1. In Vitro Studies

#### 5.1.1. Antimicrobial Activity

Effective treatment of acne requires the development of targeted antimicrobial approaches, with an emphasis on neutralizing *Cutibacterium acnes* while simultaneously limiting cytotoxicity and antimicrobial resistance. Recent advances in the design of antimicrobial peptides and nanostructured delivery systems have enabled the creation of selective agents with enhanced potency and improved safety. The following studies highlight modern strategies for achieving potent anti-*Cutibacterium acnes* activity through rational peptide design and advanced technological platforms.

In a study conducted by Hyun Kim et al. (2025) [54], the authors focused on creating short, simply structured peptides that could show targeted biological activity while remaining cost-efficient to produce. For this purpose, they prepared a set of 13-amino-acid antimicrobial peptides and assessed their performance against *Cutibacterium acnes*. The evaluation included both standard laboratory strains and strains resistant to commonly used antibiotics. To examine potential cytotoxic effect, the peptides were tested on human erythrocytes and HaCaT human keratinocyte cell lines. The safety profile was confirmed using the MTT assay. The investigation involved measuring keratinocyte viability at different peptide concentrations. Based on combined antimicrobial and cytotoxicity data, de novo anti-*Cutibacterium acnes* peptides-7 and de novo anti-*Cutibacterium acnes* peptides-10 were identified as the most promising molecules. All designed peptides displayed activity against both strains of *Cutibacterium acnes*, which indicates their potential as antimicrobial agents.

**Figure 7:**
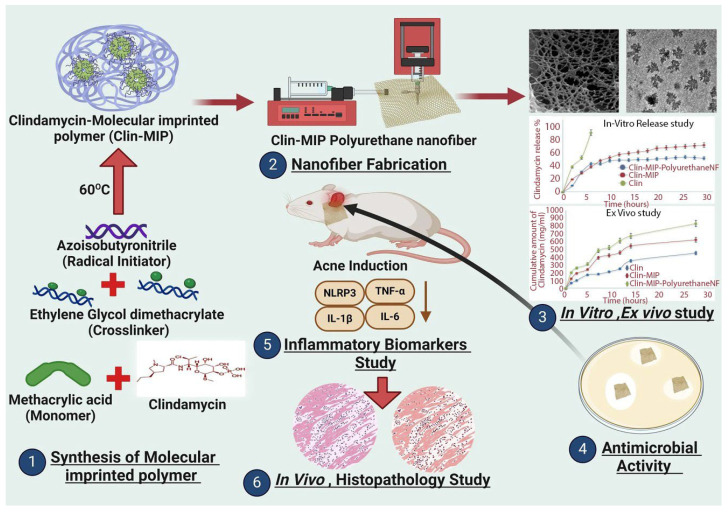
Schematic representation of the research presented by Sammar Fathy Elhabal et al. (2024) [55].

In research presented by Sammar Fathy Elhabal et al. (2024) [55], the authors focused on developing a clindamycin formulation using molecular imprinting technology. In their approach, clindamycin was first incorporated into molecularly imprinted polymeric NPs prepared by precipitation polymerization. These NPs were then integrated into polyurethane nanofibres produced through electrospinning. The antimicrobial assessment showed that the molecularly imprinted polyurethane nanofibre exhibited notable activity against every examined bacterial strain in comparison with blank clindamycin and gentamycin control. The clindamycin-loaded nanofibre demonstrated strong inhibitory effects, particularly against *Klebsiella pneumoniae*, *Proteus vulgaris*, and *Pseudomonas aeruginosa*, with inhibition zones over the ones produced by gentamycin (Figure 7).

In a study reported by Qichang Dong et al. (2023) [56], the authors introduced a structured workflow incorporating multiple deep learning models to design antimicrobial peptides that selectively target *Cutibacterium acnes* while maintaining non-haemolytic properties. From their computational library, 42 peptide candidates were chosen for synthesis and tested in vitro to assess their antimicrobial activity at different concentrations. A peptide was classified as active when its inhibitory effect on *Cutibacterium acnes* exceeded 50% at the tested dose. The evaluation identified 14 peptides with strong activity, 16 with medium activity, 4 with weak effects, and 8 without detectable antimicrobial properties. Focusing only on medium- and high-potency peptides, the design strategy achieved a success rate of 71.4%, which indicated high effectiveness.

#### 5.1.2. Effects on Sebum-Producing Cells

Sebocytes are a key regulator of acne development, as lipid overproduction and activation of inflammatory pathways contribute significantly to its pathogenesis. Approaches that directly target the regulation of cell proliferation, differentiation, and sebogenesis, in addition to antimicrobial activity, provide a comprehensive therapeutic strategy. The following studies explore how advanced delivery systems and natural or synthetic bioactive substances affect sebocyte function and lipid metabolism, both in experimental and animal models.

In the work of Wen-yu Cao et al. (2024) [57], a microneedle patch based on ALA/AgNPs@DMNs that was encapsulated into a dual drag was designed using antibacterial metal NPs together with photodynamic therapy. The concept relied upon the penetration of microneedles into the stratum corneum, making possible an enhanced delivery of drugs to be conducted on the lesion site. Experiments conducted both in vitro and in vivo demonstrated that ALA/AgNPs@DMNs possess antibacterial activity and can effectively lower inflammation. As a result, this simple but highly effective therapeutic strategy shows considerable potential for managing epidermal skin disorders.

The study conducted by Si Liu et al. (2023) [58] aimed to better understand the anti-acne effects of emodin. The investigated compound is an anthraquinone-based molecule found in nature. It has antiproliferative and anti-inflammatory actions in skin conditions. In this work, the researchers examined how emodin affects cell growth, the cell cycle, apoptosis, insulin-like grow factor (IGF-1)-caused lipid production, and *Cutibacterium acnes*-triggered inflammation in human SZ95 sebocytes. IGF-1 is considered an important factor in acne development because it increases the activity of sebaceous glands. To check how emodin influences the growth of SZ95 sebocytes, a CCK-8 test was used. The results showed that sebocyte growth clearly decreased after treatment with emodin. The study also found that emodin reduced the protein levels of PPARy, LXRα/β, and SREBP-1 in a dose-dependent way. This confirms that emodin can slow down sebocyte differentiation and limit sebum production in SZ95 cells.

Takamichi Kitano et al. (2024) [59] examined how ozenoxacin affects sebum production in hamster sebaceous glands, both in vitro and in vivo. The researchers showed that ozenoxacin reduces sebum production in hamster sebocytes that were differentiated with 5α-DHT in vitro. They also observed that, like ozenoxacin, nadifloxacin and clindamycin can lower sebum production in sebocytes stimulated by androgens, which is especially relevant during adolescence. In addition, ozenoxacin was found to exhibit insulin-induced sebum synthesis in hamster sebocytes. The results suggest that ozenoxacin and other dermal antimicrobial agents have anti-acne effects not only because they act against *Cutibacterium acnes* but also because they limit lipid production in sebocytes influenced by androgens, insulin, and IGF-1.

### 5.2. In Vivo and Clinical Findings

#### 5.2.1. Skin Compatibility

Skin compatibility and tolerability are critical factors determining long-term compliance and therapeutic success in acne management. Clinical evaluation of both systemic and topical anti-acne agents is therefore essential to ensure efficacy without causing irritation, sensitization, or pigmentary changes. The following clinical studies evaluate the safety profiles and cutaneous tolerability of established and emerging acne treatments in diverse patient populations.

Jianzhong Zhang et al. (2025) [60] explored how effective and safe sarecycline is for treating moderate-to-severe acne vulgaris. This phase 3 clinical trial was designed to provide evidence on the medicament’s efficacy and safety profile. It was a multicentre, double-blind, placebo-controlled study with parallel groups, including patients aged 9 to 45 years. The results showed that sarecycline was more effective than placebo for all measured outcomes. Participants receiving sarecycline had better reduction in inflammatory lesions on the face. The drug also worked better than placebo for non-inflammatory lesions. Sarecycline was generally well tolerated, and its safety was comparable to placebo. Rates of treatment-emergent side effects also were similar. During the study, no cases of post-inflammatory hyperpigmentation were reported.

The clinical trial by Yan Le et al. (2024) [61] investigated how effective topical minocycline foam (FMX101 4%) is for treating moderate to severe facial acne in Chinese patients. The study also included safety assessment. This was a multicentre, randomized double-blind, vehicle-controlled phase 3 study. The results showed that the number of inflammatory lesions began to decrease by week 4. This improvement was continued until the end of the treatment period. The researchers also found that FMX101 4% did not cause any meaningful photoreactive or photosensitive reactions, skin sensitization, or chronic irritation. Among patients treated with FMX101 4%, the most frequent side effects on the face were mild redness, pigmentation changes, itching, skin exfoliation, and loss of moisture.

Another clinical study by Nasrin Saki et al. (2025) [62] examined how effective intramuscular pantothenic acid injections and topical adapalene are when used together. A total of 59 patients included in the study suffered from mild-to-moderate acne vulgaris. This was a single-blind, randomized controlled trial carried out in dermatology clinics. Participants were divided into two groups. One of them used adapalene alone, and the other received adapalene and pantothenic acid injections. The results showed that both treatments led to improvement in the patients’ quality of life. The severity of acne and the number of lesions decreased. The combination therapy was no more effective than adapalene used by itself, because no notable differences was found between the two groups.

#### 5.2.2. Clinical Improvement in Acne Symptoms

Clinical improvement in acne is assessed through objective measures such as reduction in the number and severity of lesions, as well as through safety and tolerability indicators of the treatment. Modern and emerging therapeutic approaches, including bacteriophages, combination antibiotic regimens, and hormonal modulators, have demonstrated significant clinical benefits in preclinical and clinical models. The following studies document the therapeutic efficacy of these interventions through measurable improvements in acne symptoms, accompanied by favourable safety profiles.

In a study by Amit Rimon et al. (2022) [63], researchers tested whether bacteriophages applied directly to the skin could be used as a new treatment option for acne. To do this, they used a mouse model of acne. They focused on phages that specifically target *Cutibacterium acnes*. After acne-like skin lesions were induced in 38 female ICR mice, the animals were divided into two groups. One group received a topical gel containing the FD3 phage, which was applied once a day for five days. The control group was treated with the same gel without phages. The results showed that the applied phages were able to penetrate the full thickness of the mouse skin and stay active within the lesions. Tissue analysis also revealed that mice treated with the FD3 phage gel had clearly milder skin lesions, with smaller size, less swelling, and reduced surface damage compared to untreated and vehicle-only groups.

M. Kutub Udin et al. (2024) [64] investigated whether the combination of doxycycline and trifarotene is effective in treating moderate-to-severe acne vulgaris. The study included 100 patients. The treatment plan consisted of oral doxycycline (100 mg once daily) taken for 18 days each month over a period of two months. At the same time, participants applied trifarotene 0.005% cream once daily for a total of 12 weeks. The results showed that treatment with trifarotene, used either on its own or combined with doxycycline, led to clear improvements in both facial and truncal acne. The therapy was generally well tolerated, and only mild to moderate undesired effects were reported. After 12 weeks, there were meaningful reductions in lesion numbers and Investigator’s Global Assessment scores. About 35% of patients showed noticeable improvement by weeks 9–10. A reduction in *Cutibacterium acnes* growth was observed. Patient satisfaction was also positive, with 36% reporting a good treatment response.

Adelaine Hebert et al. (2020) [65] assessed the function of topical clascoterone cream 1% for people with facial acne and also evaluated safety. The authors conducted two identical phase 3 clinical trials that were multicentre, randomized, double-blind, and vehicle-controlled. Patients were assigned to two groups. One group used clascoterone 1% cream. The second one applied a vehicle cream without the active ingredient. Both trials successfully met their main study goals. After 12 weeks of treatment, a much higher number of patients using clascoterone achieved treatment success. Clascoterone was also more effective than vehicle cream in lowering inflammatory and non-inflammatory lesion counts. The treatment was well tolerated, and no important safety problems were reported during the trial.

### 5.3. Comparative Analysis of Essential Oil Nanoformulations and Conventional Treatments

Clinical response to acne is primarily reflected in a reduction in the number and severity of skin lesions, as well as the overall burden of the disease. Both novel and established therapeutic interventions, such as microbially targeted approaches, combination therapy regimens, and agents that modulate hormonal activity, have demonstrated documented benefits in experimental and clinical models. The following studies examine the efficacy of these interventions based on objective clinical markers, as well as histological findings or patient-reported outcomes.

Mohammed H. Taleb et al. (2018) [66] compared the effects of an oregano (*Origanum vulgare* L.) essential oil nanoemulsion with those of 2% erythromycin in an animal model of acne. The anti-acne activity was tested using mice in which acne-like inflammation was caused by *Cutibacterium acnes* infection. The researchers applied either the oregano oil nanoemulsion or 2% erythromycin solution directly to the infected ears of the mice. A separate group of animals received no treatment and served as a control. The findings showed that the oregano essential oil nanoemulsion reduced skin inflammation more effectively than erythromycin throughout the treatment period. Mice treated with the oregano formulation also showed lower bacterial levels, faster tissue repair, and greater overall improvement compared with those treated with erythromycin. By the end of the study, the oregano nanoemulsion achieved a much higher reduction in inflammation (more than 60%) than the antibiotic treatment (about 20%).

Farah M. Abdelhamed et al. (2022) [16] evaluated a thyme essential oil nanoemulsion as a possible new option for acne treatment. The researchers compared its effects with clindamycin using an animal model. The formulation was tested for its healing and anti-inflammatory properties in mice with acne-like skin inflammation. The results confirmed that thyme essential oil nanoemulsion showed strong antibacterial activity against *Cutibacterium acnes* and effectively reduced inflammation. Treatment with the thyme nanoemulsion led to a faster decrease in ear swelling than treatment with clindamycin. By the end of the study, mice receiving the thyme formulation showed a greater reduction in inflammation compared with the clindamycin-treated group. NF-κB levels in mouse ear tissue were measured by ELISA. Thyme nanoemulsion reduced NF-κB to values comparable with healthy mice and caused a 5-fold decrease, while clindamycin achieved only a 2.5-fold reduction.

These comparative findings suggest that nanoformulated essential oils may offer therapeutic efficacy comparable to, and in some cases exceeding, conventional acne treatments, particularly in terms of antimicrobial activity and lesion reduction. However, larger and well-designed clinical trials are still required to fully confirm whether these systems can consistently outperform established standard therapies in routine dermatological practice.

## 6. Safety, Toxicity, and Regulatory Considerations

### 6.1. Biocompatibility of Eco-Friendly Shell Materials

Biocompatibility is a fundamental criterion for shell materials used in eco-friendly nanoformulations designed for topical and biomedical use. Biopolymers of natural origin, including alginate, chitosan, and poly(ε-caprolactone), provide advantageous cell–material interactions while maintaining mechanical stability and functional efficiency. The studies that follow examine cytocompatibility and cell responses related to these sustainable shell components in core–shell nanoscale systems.

Samira Sasan et al. (2024) [67] developed a nanofibrous wound dressing. Zinc oxide NPs and *Salvia abrotanoides* essential oil were embedded in a sodium alginate outer layer. Poly(ε-caprolactone) formed the inner core. The researchers examined how L929 fibroblast cells behaved when exposed to those designed nanofibre membranes to evaluate biocompatibility. After 24 h, all tested materials sustained cell survival above 60%. After 48 h, cell viability was similar to that of the control group. This showed that the scaffolds were not toxic and allowed cells to attach and multiply. Among the tested materials, PCL/SA@SAEO showed the highest cell viability. In addition, nanofibres containing zinc oxide and *Salvia* essential oil supported better fibroblast adhesion and growth than PCL/SA fibres without these additives.

Sayna Shamszadeh et al. (2022) [68] produced chitosan–alginate core–shell particles using a coaxial electrospray technique and evaluated their biocompatibility. Cells were exposed to different amounts of chitosan, alginate, and the core–shell particles. The hemolysis test showed very good compatibility for the microparticles and for NPs at concentrations below 500 µg/mL, which matched the levels used in biological experiments. In the next step, the effect of the particles on adipose-derived stem cells was examined. Noticeable toxicity appeared after 72 h. Stem cells were treated then with NPs at concentrations higher than 500 µg/mL, which were outside the tested range.

### 6.2. Irritation Potential Reductions via Encapsulation

Skin irritation is a major obstacle to the use of essential oils for topical application, as their free form can adversely affect the skin barrier function. Encapsulation techniques, and nanoscale encapsulation in particular, have been shown to be effective in regulating the interaction between oils and skin through controlled release and limiting direct contact with the stratum corneum. The following studies investigate the effects of encapsulation on skin barrier integrity, hydration levels, and irritation-related markers.

Perla Giovanna Silva Flores et al. (2023) [9] examined how free and encapsulated essential oils from *Rosmarinus officinalis* and *Lavandula dentata* influence the barrier properties of porcine skin in an ex vivo model. The authors focused on changes in key biophysical parameters of the skin. The results showed that transepidermal water loss was higher after skin contact with emulsions containing free essential oils than after contact with nanoencapsulated oils. This indicates that non-encapsulated oils disrupt the skin barrier more strongly by increasing water permeability in the stratum corneum. In contrast, pH values remained close to those of untreated skin for both nanoformulations and emulsions. In addition, stratum corneum water loss (SCWL) increased after exposure to all tested formulations.

**Figure 8:**
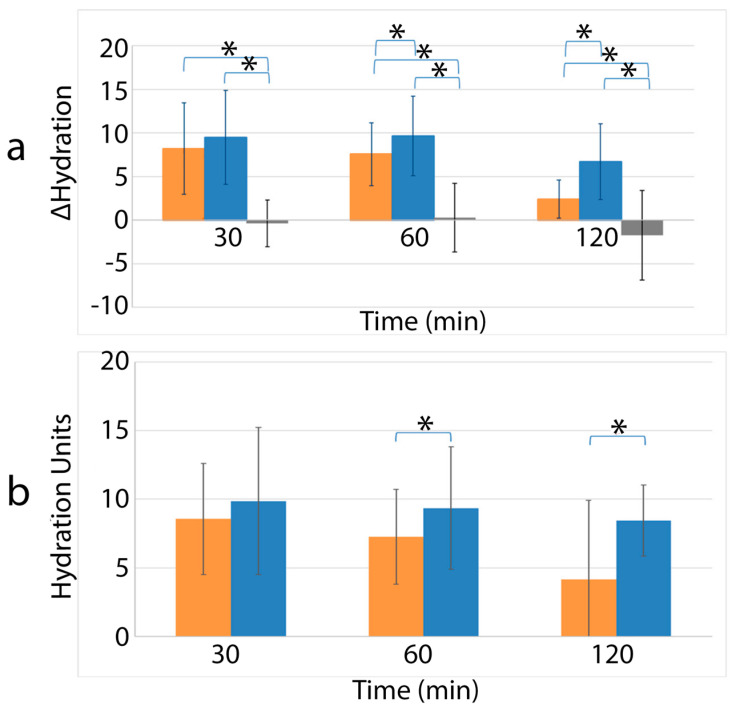
Hydration change (**a**) and normalized values (**b**) of skin treated with conventional emulsions (CEL) (■) and nanoemulsions (NEL) (■) or left untreated (■). * *p* < 0.05 [69].

According to Konstantina Flekka et al. (2024) [69], conventional emulsions and nanoemulsions containing lavender essential oil were compared. The study evaluated their effects on skin hydration and barrier function. The formulations were examined in vivo on 10 healthy volunteers. Transepidermal water loss served as a measure of skin hydration and barrier recovery. Both types of formulations visibly increased skin hydration. This effect persisted for at least two hours after application. The nanoemulsion showed a stronger moisturizing effect than the conventional emulsion. Both formulations caused a similar rise in transepidermal water loss after temporary disruption of the skin. In contrast, only the nanoemulsion lowered transepidermal water loss compared with untreated skin, indicating faster barrier repair (Figure 8).

### 6.3. Regulatory Hurdles for Natural Nano-Dermatological Products

The increasing use of nanomaterials in cosmetic formulations is being reviewed in this article, emphasizing the regulatory scrutiny surrounding their dermal application. Current safety assessment strategies, including physicochemical characterization, toxicological testing, and exposure evaluation, are also analyzed in this work. This paper highlights the role of European regulatory bodies, particularly the Scientific Committee on Consumer Safety, in determining consumer safety. Regulatory challenges mainly arise from the limited long-term toxicity data and inconsistencies in testing methodologies. Overall, the regulatory uncertainty and how it can slow innovation in nano-enabled dermatological products is analyzed [70].

Another article that reviews the European Union’s legal framework governing nano-enhanced cosmetic products under Regulation (EC) No 1223/2009 details the mandatory notification, labelling, and pre-market safety assessment requirements for nanomaterials. The authors identify regulatory hurdles related to data availability, safety dossier preparation and Scientific Committee on Consumer Safety approval timelines. Also, the complexity of the nanomaterial definitions and risk assessment protocols is highlighted as a huge barrier. This review analyzes how these regulatory demands can affect the commercialization of innovative and natural nano-cosmetics [71].

The regulatory approaches for cosmetic nanocarriers in Europe and North America are compared in this review, focusing on their impact on product development. It emphasizes how nanocarriers enhance ingredient stability and skin penetration but also raise safety and regulatory concerns. Challenges for global market access can be created due to differences in nanomaterial definitions and approval pathways. The lack of harmonized regulations is identified as a major obstacle for manufacturers as well. The paper concludes that clearer regulatory guidance is essential for wider adoption of nanotechnology in cosmetics [72].

## 7. Sustainability and Environmental Impact

### 7.1. Biodegradability of Materials

Biodegradability is a key factor in the design of sustainable nanocarriers, as it affects both their environmental behaviour and their safety in long-term topical or biomedical applications. Polymeric materials derived from renewable raw materials or microbial synthesis can be progressively degraded into harmless products, without degrading the effective delivery of bioactive substances. However, unregulated degradation may compromise structural integrity, cause premature release, or reduce functional stability during storage and application. Therefore, biodegradation must be engineered rather than assumed.

The rate of degradation can be controlled by adjusting the crosslink density, thereby reducing swelling and slowing hydrolytic or enzymatic breakdown. Mixing in more hydrophobic biodegradable segments through copolymerization or blending lowers water absorption and slows degradation. Surface treatments and protective coatings can also delay early erosion but still allow the material to break down over time. In lipid-based nanocarriers, adjusting the crystallinity of the matrix improves structural stability and helps control the release of active compounds. Using responsive linkages and multilayer shell structures can make nanocarriers more precise. These features help the carriers remain stable during use but degrade when exposed to certain physiological or environmental conditions. With these methods, biodegradable nanosystems can stabilize essential oils, exhibit strong antimicrobial activity, enable controlled release, and reduce environmental impact by breaking down safely after use.

The following studies highlight biodegradable nanosystems that simultaneously achieve essential oil stabilization, remarkable antimicrobial activity, and a reduced environmental footprint.

**Figure 9:**
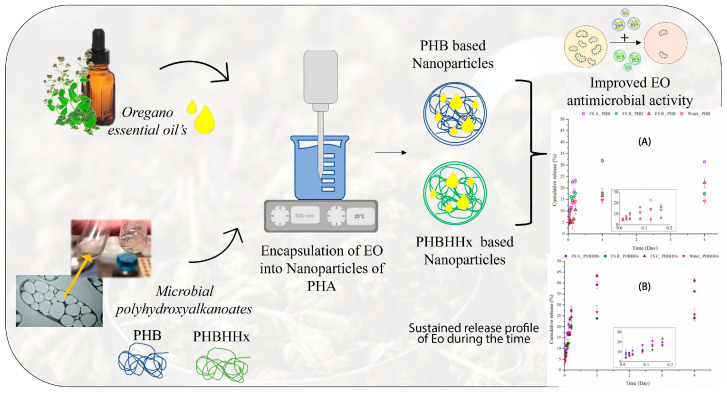
Schematic representation of the research presented by Iolanda Corrado et al., cumulative release profile of EO-loaded PHB (**A**) and PHB-HHx (**B**) [73].

In research presented by Iolanda Corrado et al. (2022) [73], the authors developed biodegradable polyhydroxyalkanoate-based NPs to encapsulate Mexican oregano essential oil, confronting the challenges of volatility and instability by leveraging a biobased delivery matrix. Essential oils were incorporated into NPs composed of polyhydroxybutyrate (PHB) and its microbial copolymer PHB-HHx via a solvent-evaporation technique. The resulting NPs displayed a narrow size distribution (150–210 nm) and achieved high encapsulation efficiency (>60%) and loading capacity (~50%), with controlled release kinetics fitting the Korsmeyer–Peppas model, indicating diffusion-driven release. Importantly, because PHBs are biodegradable polyesters produced by microbial fermentation, these nanocarriers degrade into non-toxic byproducts under appropriate conditions, reducing environmental persistence relative to synthetic polymers. Both types of essential oil-loaded NPs retained antimicrobial activity against *Micrococcus luteus*, with PHB-HHx carriers showing enhanced efficacy compared with the free oil, demonstrating that biodegradable polymer matrices can support stable and functional essential oil delivery while meeting environmental sustainability goals (Figure 9).

Also, in a study by Roxana Gheorghita Puscaselu et al. (2024) [74], researchers investigated the synthesis of biopolymer-based nanocarriers for essential oil delivery with a focus on biodegradability and material safety. Using biopolymeric matrices derived from natural polysaccharides, the team fabricated core–shell constructs that exhibited sustained degradation in aqueous environments without generating harmful byproducts. Biodegradation assessments coupled with physicochemical analyses confirmed that the nanocarriers gradually lost structural integrity over time, indicating controlled decomposition under simulated physiological conditions. Antimicrobial assays demonstrated that these biodegradable carriers effectively released encapsulated essential oil activities and maintained antimicrobial activity against model Gram-positive and Gram-negative strains.

### 7.2. Green Chemistry in Synthesis

The application of green chemistry principles to the synthesis of nanomaterials promotes the use of plant-derived extracts and essential oils as natural reducing and stabilizing agents, reducing the need for toxic chemical reagents. These bioinspired approaches allow the production of metal and metal oxide NPs with controlled physicochemical characteristics and remarkable biological activity. The following studies highlight how green synthesis contributes to the sustainability and functional performance of nanocarriers for biomedical applications.

In their research, Fahimeh Golzarnezhad et al. (2025) [75] explored a green chemistry approach to synthesize ZnO NPs using aqueous leaf extract of *Cymbopogon olivieri* as a natural reducing and stabilizing agent. Characterization via UV-vis spectroscopy, X-ray diffraction, and FTIR spectroscopy confirmed the formation of highly crystalline ZnO NPs with an average size of approximately 28 nm. The authors underscored that using plant metabolites as biological reductants eliminated the need for hazardous chemical reagents typically used in conventional metal oxide nanoparticle synthesis. Antimicrobial assessments revealed that the biogenic ZnO NPs exhibited effective inhibitory activity against both Gram-positive and Gram-negative bacteria, demonstrating potential for eco-friendly biomedical and antimicrobial applications.

In a comparable study, Mir-Hassan Moosavy et al. (2023) [28] reported the green synthesis of gold and silver NPs using *Mentha spicata* essential oil as both the reducing and capping agent. The essential oil, rich in carvone and limonene, facilitated the formation of spherical gold and silver NPs under ambient conditions without the use of synthetic reducing chemicals. Characterization by TEM, DLS, and FTIR confirmed nanoparticle formation with sizes of approximately 19.6 nm (Au) and 24 nm (Ag). Both AuNPs and AgNPs exhibited significant antimicrobial and antioxidant activities, with AgNPs showing superior antibacterial efficacy against common bacterial strains. The authors emphasized that this green synthesis route harnessed natural phytochemicals as eco-friendly reagents, reducing dependence on non-renewable chemical inputs and improving the sustainability profile of nanoparticle production for potential biomedical applications.

### 7.3. Reduced Reliance on Synthetic Antimicrobials

Encapsulation of essential oils in nanostructured systems offers a viable strategy to reduce the dependence on synthetic antimicrobials, while enhancing their stability and bioactivity. Through controlled release and improved bioavailability, these natural compounds can achieve antimicrobial efficacy comparable to conventional antibiotics. The following studies highlight the role of nanocarriers in substituting or complementing synthetic antimicrobials with more ecological and safe alternatives.

In research presented by Maryam Fakhariha et al. (2025) [8], the authors investigated nanoencapsulation strategies to enhance the antimicrobial efficacy of natural essential oils, therefore presenting alternatives to conventional synthetic antimicrobial agents. Thyme and sage essential oils were encapsulated in hollow polymeric nanospheres, and the resulting formulations were evaluated for antimicrobial activity against *Escherichia coli* and *Staphylococcus aureus*. Encapsulated thyme essential oil exhibited potent antibacterial activity with minimal inhibitory concentrations as low as 2–4 µL/mL, which was significantly enhanced compared to non-encapsulated oil, likely due to sustained release from the porous silica matrix. The controlled-release profiles helped maintain antimicrobial effects over time without relying on synthetic compounds.

In a study by Israa Al-Ogaidi et al. (2022) [76], biodegradable nanoliposomes encapsulating essential oils were developed as antimicrobial agents for topical creams and gels, providing a natural alternative to conventional synthetic antimicrobials. The liposomal encapsulation system demonstrated near-complete essential oil loading, increased stability, and antimicrobial activity against bacterial strains comparable to that of the antibiotic kanamycin. By substituting synthetic solvents and antimicrobial substances with biodegradable lipids and natural essential oils, the authors achieved effective antimicrobial formulations that correspond with sustainable therapeutic design principles.

## 8. Challenges and Future Perspectives

**Figure 10:**
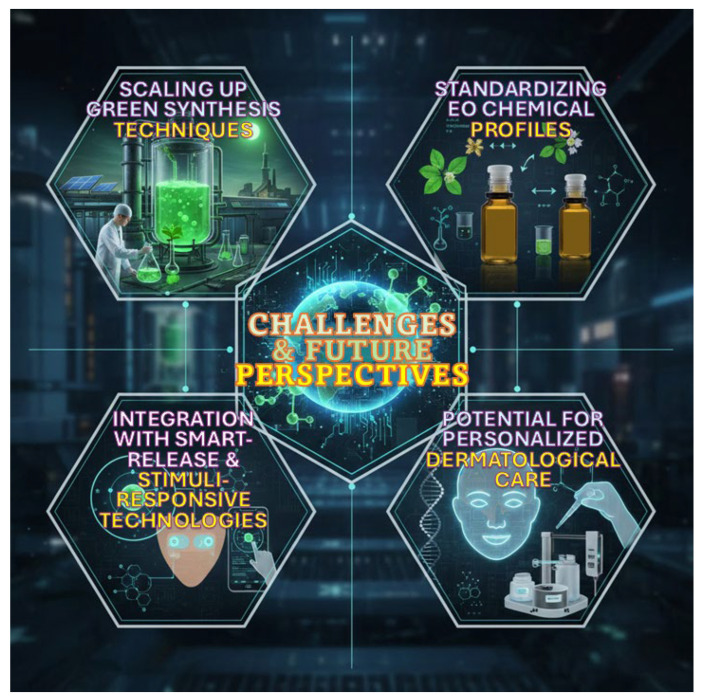
Schematic representation of challenges and future perspectives related to biodegradable polymeric core/shell nanoformulations.

Although biodegradable core–shell nanocarriers represent a leap forward in acne therapy, the instability of essential oil payloads hinders their clinical adoption, manufacturing variability, and presents a lack of robust data on long-term skin biocompatibility. These limitations may slow down their commercial development, as long-term safety studies are essential before regulatory approval and widespread clinical use (Figure 10).

### 8.1. Scaling up Green Synthesis Techniques

This chapter outlines how biological and plant-based methods for synthesizing NPs can present major problems when transitioning from lab to industrial scale. The need for reliable, cost-effective strategies to produce high yields of green nanomaterials without toxic chemicals is analyzed, as well as challenges such as raw material variability, process optimization, and manufacturing costs. The chapter stresses that addressing these issues is essential for commercial adoption of sustainable nanoformulation methods [77].

Moreover, this review emphasizes the promise of green synthesis routes using herbs, fungi, bacteria, and waste biomass as alternatives to traditional hazardous methods. Green synthesis may offer environmental benefits like lower energy use and reduced pollution, but scalability remains limited by inconsistent raw sources and process control. This paper highlights the challenges related to reproducibility of nanoparticle properties at larger scales and the need for standardized production techniques. It underscores that achieving scalable green production is crucial for broad industrial applications. It also points to how these methods align with sustainability goals across sectors [78].

This review highlights the significant challenges in moving green NPs from the laboratory to realistic large-scale healthcare applications. A key challenge is ensuring consistent product quality and precisely controlling critical parameters during scale-up, such as size and surface characteristics. The authors point out that many green approaches lack comprehensive engineering design and process control strategies, which are essential for industrial production. In addition, shortcomings in the regulatory framework further hinder commercialization. This article concludes that addressing the challenges of scale-up requires coordinated interdisciplinary collaboration between chemistry, engineering, and regulatory sciences [79].

### 8.2. Standardizing Essential Oil Chemical Profiles

The standardization of the chemical profiles of essential oils is a significant challenge, as genetic, geographical and technological factors influence their composition, as well as the contribution of minor components. Recent approaches combining high-resolution analytical techniques with chemometric and computational tools allow for reliable differentiation and authentication of oils. However, harmonized protocols and a deeper understanding of the variability factors are required for effective quality assurance and reproducibility of their bioactive properties.

Freddy A. Bernal et al. (2022) [80] investigated variations in the essential oil composition of *Magnolia grandiflora* leaves. The authors combined GC-MS profiling with chemometric methods. The analysis revealed differences among the samples, which allowed the authors to differentiate two separate chemical profiles. Samples in the first group were characterized by high levels of α- and β-pinene. Those in the second group showed increased contents of β-elemene, bicyclogermacrene, and germacrene D. The separation of these profiles was supported by several statistical techniques applied. Those approaches included full chromatography fingerprints and the relative concentrations of individual compounds. The study further demonstrated that sample differentiation within each group and between the two profiles was influenced not only by major constituents but also by minor components. However, the role of environmental conditions in shaping these compositional differences remains unclear and should be explored in future research to strengthen quality control approaches.

CH Ratnasekhar et al. (2025) [81] proposed a fast and non-destructive method to verify the authenticity of essential oils from *Menta* and *Ocimum* species. The approach combined Fourier Transform Near-Infrared spectroscopy with machine learning, while GC-Flame Ionization Detector and GC-MS were used to identify key chemical components. Distinct chemical profiles were observed for individual species. *Mentha arvensis* contained mainly menthol and methyl acetate. *M. spicata* was rich in carvone and limonene. *M. piperita* showed high levels of menthol, menthone, and menthofuran. *M. citrata* was dominated by linalool and linalyl acetate. For *Ocimum* oils, *O. basilicum* was characterized by eugenol and β-elemene; *O. sanctum* by eugenol and caryophyllene; and *O. kilimandscharicum* by a high camphor content. After removing irrelevant variables, the machine learning morels achieved very high accuracy in distinguishing oil species, their mixtures, and samples adulterated with vegetable oils, highlighting their usefulness for essential oil quality control.

Beyond basic compositional profiling, additional strategies are required to address intrinsic variability of essential oils. These include chemotype selection, controlled cultivation conditions, and the use of certified botanical raw materials to minimize fluctuations in key constituents. Fractionaction and reconstitution approaches have also been explored to adjust the concentration of dominant bioactives to predefined ranges. From an analytical perspective, standardized detection relies primarily on validated GC-MS and GC-FID protocols with internal standards, calibration curves and retention index matching for accurate compound identification.

### 8.3. Integration with Smart-Release and Stimuli-Responsive Technologies

The incorporation of essential oils and other bioactive compounds into “smart” controlled-release systems that respond to stimuli, such as pH, temperature, or redox environment, presents significant technological challenges. Although such platforms allow for spatiotemporal controlled release and increased therapeutic precision, their complex architecture makes scaling, stability, and reproducibility difficult. A major challenge lies in the limited size and variability of physiological triggers within the skin. Although acne lesions may exhibit slightly reduced pH and increased oxidative stress, these changes are not uniform and can fluctuate overtime, which complicates the design of systems that rely on precise stimulus thresholds. Materials engineered to respond under well-defined laboratory conditions may therefore show weakened or inconsistent activation in vivo. Structural limitations further complicate integration: in core–shell nanoformulations, the responsive component must remain stable during storage while retaining sensitivity after topical application. Further research is needed to simplify the design and reliably incorporate stimulus-responsive mechanisms into viable and clinically applicable nanoplatforms.

Siyuan Deng et al. (2021) [82] reported the development of redox-responsive core–shell nanohydrogel systems. Those nanocapsules (NanoCs) were designed for intracellular delivery. The structure consisted of a hydrophilic core crosslinked via disulfide bonds and a hydrophobic shell formed through interfacial Michael addition. The authors showed that these nanocapsules effectively protected the encapsulated model protein and preserved its activity under physiological conditions. In a reducing environment, rapid payload release occurred due to redox-induced degradation system. These findings indicate that redox-responsive NanoCs are promising carriers for controlled and cell-specific drug delivery.

Elif Gulin Ertugral-Samgar et al. (2023) [83] developed a drug-loaded nanoparticle system combined with a thermosensitive hydrogel scaffold as a delivery system for controlled drug release. The study focused on achieving a slower and more predictable release profile. Curcumin release was evaluated from free NPs and from NPs embedded in the hydrogel. Experiments were conducted at body temperature and at pH 7.4 and 5.5. At pH 7.4, drug release remained minimal over four days. At pH 5.5, both systems showed sustained release. Free NPs release nearly four times more curcumin than the hydrogel-based formulation.

In the study by Renata Pinho Morais et al. (2023) [84], the integration of pH-responsive behavior into hybrid core–shell NPs revealed several formulation challenges. The release of ethyl acetate fraction (EAF) depended strongly on environmental pH, yet the expected behaviour based on polymer pKa values was not fully observed. For chitosan-coated hybrid NPs (SNP-EAF-CH), a higher release at pH 5.4 was expected due to chitosan chain expansion, but the system instead showed limited release. This was attributed to low EAF solubility and possible interactions between tannins and amino groups of chitosan. This indicates that polymer responsiveness alone does not determine release performance. In addition, polymer coatings were required to block silica pore entrances in order to prevent premature diffusion, but this structural barrier also had to allow stimulus-dependent release at pH 7.4. FTIR analysis demonstrated that silica core dissolution was accelerated under basic conditions, contributing to increased release, whereas acidic conditions preserved core integrity and reduced EAF diffusion. These findings show that smart release integration depends on the balance between polymer ionization, silica stability and EAF solubility. The system must support pore blocking under skin surface conditions while enabling structural changes and core dissolution in the follicular environment.

### 8.4. Potential for Personalized Dermatological Care

Personalized dermatological care is emerging as a promising but challenging direction, based on the use of molecular biomarkers and advanced nanodelivery systems to tailor therapy to the patient’s individual pathophysiological profile. Despite the potential offered by stimulus-responsive nanocarriers and artificial intelligence for dynamic and targeted control of active agent release, challenges remain related to clinical validation, data complexity, and translatability of these technologies to daily practice.

This review highlights the importance of personalized medicine in dermatology, where molecular biomarkers are used to tailor treatments for conditions such as psoriasis, atopic dermatitis, and alopecia, improving the accuracy of treatment. It is highlighted that the identification of specific biomarkers for each condition allows physicians to tailor treatments to the individual pathophysiological profile of patients, leading to better outcomes. Emphasis is given on the integration of technologies such as artificial intelligence and nanotechnology, which enhance the utility of biomarkers in clinical practice. The article argues that leveraging patients’ unique molecular characteristics allows personalized dermatological strategies to maximize efficacy and reduce adverse effects. Overall, it presents personalized dermatology as a dynamically developing field, driven by molecular diagnostics and advanced technologies [85].

This review explores how advanced nanocarrier systems, including stimulus-responsive delivery vehicles, can enable controlled and targeted release of active ingredients in skincare products, paving the way for personalized dermatological therapies. It is emphasized that tailoring the properties of nanocarriers, such as pH or temperature sensitivity, enables formulations to respond dynamically to individual skin conditions. Furthermore, the authors highlight the potential for integrating digital technologies and artificial intelligence to further optimize personalized delivery strategies. Such “smart” systems could regulate the release of therapeutic agents in real time, based on feedback from the skin, increasing efficacy and reducing side effects. Overall, the work highlights the critical role of nanotechnology in tailoring dermatological interventions to the individual needs of patients [86].

There is a clear need for more interdisciplinary research, particularly at the intersection of dermatology and materials science. The complex structure and barrier properties of human skin pose significant challenges for the effective delivery of bioactive compounds, whether for therapeutic or cosmetic purposes. Materials science provides tools to design advanced carriers—such as NPs, hydrogels, or core–shell systems—that can protect sensitive molecules, control their release, and enhance penetration through specific skin layers. At the same time, dermatology offers essential insights into skin physiology, pathology, and cellular responses, which are critical for ensuring safety and efficacy. By combining these fields, researchers can develop formulations that are not only functionally optimized but also biocompatible and tailored to specific dermatological applications. Such interdisciplinary approaches are vital for translating novel materials into clinically and commercially relevant products, ultimately improving patient outcomes and expanding the possibilities for personalized skincare and treatment strategies.

## 9. Conclusions

Eco-friendly core–shell nanoformulations were shown to improve the physicochemical stability, dermal performance, and therapeutic potential of essential oils for acne and sebum control. The systems reached encapsulation efficiencies above 70%, reduced volatilization and oxidative degradation compared to free essential oils, and increased aqueous dispersibility. This allowed stable incorporation into topical formulations. In vitro release studies indicated controlled, sustained release with a lower initial burst and prolonged diffusion over several hours. These properties support extended antimicrobial and sebum-regulating effects.

Biodegradable polymers, plant-based lipids, and polysaccharides used as core and shell materials provided favourable skin biocompatibility and low irritation, supporting their suitability for dermal delivery. The core–shell architecture facilitated improved retention of active compounds within the pilosebaceous unit, which is the main site of acne-related microbial colonization and sebum accumulation. Green synthesis approaches, such as solvent-minimized processing and plant-mediated fabrication, reduced environmental impact without compromising functional properties, aligning with green chemistry objectives.

Overall, the results indicate that sustainable nanocarrier design can enhance essential oil stability, control release kinetics, improve skin compatibility, and reduce environmental impact. Eco-friendly core–shell nanoformulations offer a rational and scalable alternative to conventional synthetic anti-acne agents, providing multifunctional, environmentally responsible, and clinically relevant delivery systems.

## Figures and Tables

**Table 1:** Sporozoite inhibition after treatment with oregano essential oil (OEO), sage essential oil (SEO), and thyme essential oil (TEO) at various concentrations [15].

Time Point	Pretreatment	100 μg mL^−1^	50 μg mL^−1^	20 μg mL^−1^	5 μg mL^−1^
2 hpi	OEO	82.8 ± 6.95	15.5 ± 37.1	0 ± 0	0 ± 0
	TEO	81.3 ± 14.1	62.8 ± 24.6	62.9 ± 20.3	24.1 ± 57.1
	SEO	72.2 ± 18.4	33.0 ± 44.8	11.6 ± 35.0	5.25 ± 14.6
24 hpi	OEO	92.9 ± 6.9	81.5 ± 25.6	38.1 ± 13.1	33.3 ± 66.7
	TEO	90.8 ± 17.9	73.1 ± 1.6	49.6 ± 54.9	67.4 ± 18.5
	SEO	89.6 ± 9.4	32.5 ± 65.1	31.6 ± 63.3	38.5 ± 42.5

**Table 2:** Biological activities of essential oils applied in acne treatment [7,11,12,14,17,18].

Essential Oil	Reported Biological Activity
*Citrus reticulata Blanco* oil	Antimicrobial activity against *Cutibacterium acne*, *Esterichia coli*, *Staphylococcus aureus*, and *Bacillus subtilis*.
*Zanthoxylum schnifolium Sieb. Et Zucc* oil	Antimicrobial activity against *Staphylococcus epidermidis.*
Tea tree oil	Antimicrobial activity against *Staphylococcus epidermidis*, *Cutibacterium acnes*, and *Lactobacillus plantarum*.
*Citrus* oil	Anti-inflammatory activity.
*Origanum vulgare* (oregano) oil	Antioxidant and antimicrobial activity.
*Salvia fructicosa* (sage) oil	Antioxidant and antimicrobial activity.
*Thymus vulgaris* (thyme) oil	Antioxidant and antimicrobial activity against *Cutibacterium acne* and *Staphylococcus epidermidis*.
*Saposhnikovia divaricata* oil	Anti-inflammatory activity.
*Leonotis nepetifolia* oil	Anti-inflammatory activity.

**Table 3:** The polymeric nanoencapsulation advantages and limitations [25,26,27,37,38,39,40].

Type of Polymeric Nanocapsule	Examples of Polymers	Main Advantages	Main Limitations
Natural polymer–based nanocapsules	Chitosan, alginate, starch derivatives, gelatin, zein	High biocompatibility and biodegradability	Batch-to-batch variability
		Derived from renewable resources	Lower mechanical strength
		Often possess intrinsic bioactivity (e.g., antimicrobial, mucoadhesive)	High swelling in aqueous media
		Suitable for dermal and biomedical use	Sensitive to pH and ionic strength
			Possible uncontrolled release without crosslinking
Synthetic biodegradable polymer nanocapsules	PLA, PCL, PLGA	High structural stability	Often hydrophobic (limited compatibility with some actives)
		Tunable degradation rate	Possible use of organic solvents during fabrication
		Controlled and predictable release kinetics	Slower biodegradation in some cases
		Good storage stability	

**Table 4:** Four eco-friendly core–shell nanoformulations evaluated based on raw materials, encapsulation efficiency, release kinetics, skin biocompatibility, and scalability.

Criteria	Polymeric Nanocapsules	Lipid-Based Nanocarriers	Polysaccharide-Based Nanocarriers	Hybrid & Bioinspired Nanostructures
Raw Material Characteristics	Biodegradable polymers (e.g., PCL, ethylcellulose, chitosan) forming a polymeric matrix/shell	Natural lipids (solid + liquid) forming a lipid matrix core; biodegradable and inherently skin-compatible	Polysaccharides (alginate, chitosan, pectin) that self-assemble into shells via electrostatic or ionic crosslinking	Plant-derived phytochemicals used as reducing and capping agents to produce metal/metal oxide NPs (e.g., Ag, ZnO) with eco-friendly synthesis
	May require organic solvents or surfactants	Often processed via high-pressure homogenization or sonication	Water-based processing	Bioinspired interfaces
Encapsulation Efficiency	Moderate–High:	High:	Very High:	Not classic encapsulation
	~60–95% depending on polymer type and formulation method	Typically ~80–90% for lipophilic drugs and antioxidants due to good affinity with lipid matrix	~90–95% reported for hydrophobic actives like curcumin (alginate)	Metal NPs do not encapsulate actives in the same way, instead provide capped/functionalized surfaces with high reactive payloads
	Effective for essential oils and lipophilic actives		Strong electrostatic capture in composite systems	
Release Kinetics	Sustained, Diffusion-Controlled/Biphasic:	Sustained with Matrix Retention:	Controlled & Responsive:	Functional Release via Ion/Surface Activity:
	Initial burst (surface-associated fraction) followed by slow polymer-controlled diffusion over hours to days	Gradual release from lipid matrix	Governed by polymer swelling, ionic exchange, and diffusion	Not diffusion through a polymer shell—biological action arises from surface ion release (Ag^+^, Zn^2+^) or ROS generation
		Matrix crystallinity and composition modulate rate	Sustained release over 24 h+	
		Minimal burst	Responsive to pH/ionic strength	
Skin Biocompatibility	Good–Very Good:	Very Good:	Excellent:	Variable Good:
	Biodegradable polymers generally well tolerated	Lipid composition mimics skin lipids	Natural polysaccharides with strong cytocompatibility (HaCaT, fibroblasts)	High biocompatibility at safe concentrations
	Reduced irritation compared with free actives	Enhanced tolerability especially for irritant actives (e.g., retinoids)	Often anti-inflammatory	Green synthesis reduces toxic surface residues
	Chitosan adds mucoadhesion			Cytotoxicity must be validated per material
Interaction with Skin Barrier	Moderate penetration	Enhances stratum corneum lipid fluidization	Strong adhesion to skin surfaces	Functional effects often at or near surface
	Appropriate for sustained topical action	Superior penetration into sebaceous layers	Effective accumulation in upper layers	Dependent on nanoparticle size and ion release dynamics
	Size can be tuned for follicular targeting		Variable penetration depth	
Scalability	Nanoprecipitation, ionic gelation, interfacial polymer deposition (scalable)	High-pressure homogenization, probe sonication (well-established industrial methods)	Room temperature assembly	Green phytofabrication scalable
			Spray-drying	Requires biological extract standardization
			Simple electrostatic complexation	

## Data Availability

No new data were created or analyzed in this study.

## Abbreviations

The following abbreviations are used in this manuscript:

DLS	Dynamic light scattering
DSC	Differential scanning calorimetry
FTIR	Fourier transform infrared spectroscopy
GC	Gas Chromatography
MS	Mass Spectrometry
MBC	Minimum bactericidal concentration
MIC	Minimum inhibitory concentration
MTT	3-(4,5-dimethylthiazol-2-yl)-2,5-diphenyltetrazolium bromide
NLC	Nanostructured lipid carriers
NPs	Nanoparticles
PLA	Polylactide acid
PLGA	Poly(lactide-co-glycolide)
PCL	Poly(ε-caprolactone)
PPAR	Peroxisome proliferator-activated receptor
SEM	Scanning electron microscopy
SLN	Solid lipid NPs
TEM	Transmission electron microscopy
TGA	Thermogravimetric Analysis
UV-Vis	Ultraviolet-visible spectroscopy
XRD	X-ray diffraction
